# Gender- and Obesity-Specific Association of Co-Exposure to Personal Care Product and Plasticizing Chemicals and Short Sleep Duration among Adults: Evidence from the National Health and Nutrition Examination Survey 2011–2016

**DOI:** 10.3390/toxics12070503

**Published:** 2024-07-11

**Authors:** Francis Manyori Bigambo, Jian Sun, Chun Zhu, Songshan Zheng, Yang Xu, Di Wu, Yankai Xia, Xu Wang

**Affiliations:** 1 Department of Endocrinology, Children’s Hospital of Nanjing Medical University, Nanjing 210008, China; 2 Department of Emergency, Pediatric Intensive Care Unit, Children’s Hospital of Nanjing Medical University, Nanjing 210008, China; 3 Department of Child Health Care, Women’s Hospital of Nanjing Medical University, Nanjing Maternity and Child Health Care Hospital, Nanjing 210004, China; 4 Department of Nursing, Nanjing Liuhe District Hospital of Traditional Chinese Medicine, Nanjing 211500, China; 5 State Key Laboratory of Reproductive Medicine, Center for Global Health, School of Public Health, Nanjing Medical University, Nanjing 211166, China; 6 Key Laboratory of Modern Toxicology, Ministry of Education, School of Public Health, Nanjing Medical University, Nanjing 211166, China

**Keywords:** short sleep duration, personal care product and plasticizing chemicals, PCPPCs, co-exposure, gender, obesity status

## Abstract

There is limited evidence about the gender- and obesity-specific effects of personal care product and plasticizing chemicals (PCPPCs) on short sleep duration in adults. We evaluated the gender- and obesity-specific association of co-exposure to PCPPCs and short sleep duration among adults aged 20–60 years using the National Health and Nutrition Examination Survey (NHANES) 2011–2016, a secondary data source from the United States. Seventeen PCPPCs, including five phenols, two parabens, and ten phthalates, were detected, and sleep duration was assessed among 3012 adults. Logistic regression, weighted quantile sum (WQS) regression, and Bayesian kernel machine regression (BKMR) were employed. We found that bisphenol A (BPA), mono (caboxy-isooctyl) phthalate (MCOP), and mono (3-carboxypropyl) phthalate (MCPP) were consistently positively associated with short sleep duration in both females and males regardless of obesity status, except for BPA with general obesity. In particular, mono benzyl phthalate (MBzP) revealed a positive association in females, mono (2-ethyl-5-oxohexyl) phthalate (MEOHP) revealed a positive association in males, and MiBP revealed a positive association in abdominal obesity. Similar associations were observed in the mixture. Our study highlights that PCPPCs are independently associated with an increasing risk of short sleep duration in adults both individually and as a mixture; however, gender- and obesity-specific differences may have little effect on certain individual PCPPCs on short sleep duration.

## 1. Introduction

Sleep is among the four human necessities for life apart from food, water, and air. For the best health, adults aged 20–60 years old are advised to sleep at least 7 h every night [1]. Sleeping less than 7 h every night is termed as a short sleep duration [1]. The prevalence of short sleep duration has increased from 31% in 2010 to 36% in 2018 among working adults in the US, while the highest prevalence was observed in people working in the military and protective services (50%), healthcare settings (45%), transportation (41%), and the manufacturing of goods (41%) [2].

Short sleep duration has been associated with adverse health effects such as cognitive impairment that may ultimately raise the chance of traffic accidents, occupational accidents, medical mistakes, and decreased work productivity in the community [3]. Numerous epidemiological studies have also documented short sleep duration to be associated with weight gain and obesity, diabetes, stroke, coronary heart disease, mental health, and death [4,5].

Studies have explored the association between certain personal care product and plasticizing chemicals (PCPPCs) such as phthalates, phenols, and parabens with sleep problems [6,7,8,9]; however, these studies did not differentiate their findings based on gender- or obesity-specific factors. Research evidence has reported significant gender differences in poor sleep quality among adults. In particular, females are more likely to have poor sleep quality compared to males [10,11]. Other studies have linked short sleep duration with obesity among females [12,13,14]. Kishman and colleagues have linked time in bed, total sleep time, sleep efficiency, and wake after sleep with body composition [15]. Furthermore, studies have indirectly linked bisphenol A (BPA) to suboptimal sleep through its obesogenic features [16,17], as obesity has been associated with short sleep duration [18].

In a few studies that have investigated the association of certain PCPPCs with sleep problems in females, the focus was only on phthalates [19,20,21], a kind of PCPPC, leaving a gap to other types of PCPPCs such as phenols and parabens. For example, mono (2-ethyl-5-carboxypentyl) phthalate (MECCP), mono-ethyl phthalate (MEP), mono benzyl phthalate (MBzP), and mono-n-butyl-phthalate (MnBP) were positively associated with short sleep duration in females. The mixture of phthalates was also positively related to short sleep duration in both the weighted quantile sum (WQS) regression and Bayesian kernel machine regression (BKMR) models [21]. Other studies have also linked phthalates with poor sleep quality in midlife women [19,20]. On the other hand, phenols such as TCS have been associated with short sleep duration in females [22]. The association between PCPPCs such as phthalates, phenols, and parabens and short sleep duration in males is limited because of data scarcity, which paves the way for further investigation.

Moreover, studies that explored the effect of PCPPCs on sleep problems irrespective of gender- and obesity-specific associations have shown that bisphenol A (BPA) [6,9] and triclosan (TCS) [9] were associated with inadequate sleep among adults. On the contrary, Shiue found no association between exposure to BPA, TCS, and parabens with sleeping disorders such as waking up at night, being unrested during the day, leg jerks, and leg cramps while sleeping [8]. The discrepancy in the results may be attributed to differences in the outcomes examined. Another study has shown that exposure to phthalates and their metabolites such as mono carboxy nonyl phthalate (MCNP), mono isobutyl phthalate (MiBP), mono-carboxyoctyl phthalate (MCOP), and di(2-ethyhexy) phthalate (DEHP) metabolites were associated with short sleep duration among adolescents [7].

Certain PCPPCs such as phthalates have been linked to the distraction of neuronal circuitry and the inhibition of the development of a hormonal-mediated mechanism that controls growth and sleep [7]. In addition, it is plausible that endocrine-disrupting chemicals including PCPPCs such as phthalates, phenols, and parabens might be linked with short sleep duration by disturbing the secretion of growth hormones that regulate the sleep/wake state [23]. PCPPCs might disrupt thyroid function and cause short sleep duration as well, since the disruption of thyroid function has been linked to short sleep duration [24]. Further, PCPPCs can disrupt the melatonin pathway, the hormone that plays a key role in the sleep/wake cycle, leading to sleep disturbance. Also, PCPPCs may be associated with short sleep duration through their ability to disrupt the central nervous system, as sleep is regulated by the central nervous system [25,26].

Due to scarce data on the gender- and obesity-specific association between PCPPCs and short sleep duration in adults, it is worth investigating the influence of gender and obesity status on the effect of PCPPCs on short sleep duration. To our knowledge, this is the first study to investigate the gender- and obesity-specific association between co-exposure to PCPPCs, including phthalates, phenols, and parabens, with short sleep duration among adults. Logistic regression, WQS regression, and BKMR models were used to examine the associations.

## 2. Materials and Methods

### 2.1. Study Population

The National Health and Nutrition Examination Survey (NHANES) is a continuing research program since 1999 under the National Center for Health Statistics (NCHS) aimed at evaluating the health and nutritional conditions of the children and adults of the United States (US) population through interviews and physical examinations [27]. The survey is designed to include oversampling, stratification, and clustering sampling methods. In the current study, we used six years of publicly available data from the NHANES 2011–2012, 2013–2014, and 2015–2016. The subjects were people aged from 20 to 60 years. Of the 29,902 participants recruited in the NHANES cycles of 2011–2016, 5800 participants had complete information on sleep hours in the sleep disorder questionnaires and PCPPCs such as phthalates, phenols, and parabens. Among participants aged 20–60 years with completed information on sleep hours and PCPPCs, we excluded those with missing information on covariates such as the family income to poverty ratio, serum cotinine, waist circumference, BMI, and underweight. Finally, 3012 participants remained in the analysis, as illustrated in Figure S1. The NCHS Research Ethics Review Board approved this study (Continuation of Protocol #2011-17) and informed consent was obtained from each participant.

### 2.2. Biomeasure of Personal Care Product and Plasticizing Chemicals (PCPPCs)

Urine samples were collected from the subjects at the mobile examination center by laboratorians, and stored frozen in polypropylene vials below 20 °C until transferred to the National Center for Environmental Health, Centers for Diseases Control and Prevention, Atlanta, Georgia, for analysis. Urine samples of 17 PCPPCs including 5 phenols, namely, benzophenone-3(BZP), bisphenol A (BPA), triclosan (TCS), 2, 5-dichrolophenol (2, 5-DCP), and 2, 4-dichlorophenol (2, 4-DCP), and 2 parabens, namely, methylparaben (MeP) and propylparaben (PrP), were quantified by the online solid-phase extraction linked to high-performance liquid chromatography isotope dilution tandem mass spectrometry, and 10 phthalates or metabolites, including mono (carboxy isononyl) phthalate [MCNP], mono (carboxy-isooctyl) phthalate [MCOP], mono (2-ethyl-5-carboxypentyl) phthalate [MECPP], mono-n-butyl-phthalate [MnBP], mono (3-carboxy propyl) phthalate [MCPP], mono-ethyl phthalate [MEP], mono (2-ethyl-5-hydroxyhexyl) phthalate [MEHHP], mono isobutyl phthalate [MiBP], mono (2-ethyl-5-oxohexyl) phthalate [MEOHP], and mono benzyl phthalate (MBzP) were quantified by high-performance liquid chromatography–electrospray ionization tandem mass spectrometry (HPLC-ESI-MS/MS). The lower limit of detection (LOD) for each PCPPC was BZP (0.4 ng/mL), BPA (0.2 ng/mL), TCS (1.7 ng/mL), 2, 5-DCP (0.1 ng/mL), 2, 4-DCP (0.1 ng/mL), MeP (1.0 ng/mL), PrP (0.1 ng/mL), MCNP (0.2 ng/mL), MCOP (0.3 ng/mL), MECPP (0.4 ng/mL), MnBP (0.4 ng/mL), MCPP (0.4 ng/mL), MEP (1.2 ng/mL), MEHHP (0.4 ng/mL), MiBP (0.8 ng/mL), MEOHP (0.2 ng/mL), and MBzP (0.3 ng/mL). The value < LOD was imputed by <LOD divided by the square root of two [28]. Urine creatinine and serum cotinine were quantified by the Beckman Synchron CX3 Clinical Analyzer and the isotope-dilution high-performance liquid chromatography/atmosphere pressure chemical ionization tandem mass spectrometric method, respectively. More information about the laboratory procedures can be obtained from the NHANES website [29,30,31,32,33,34,35].

### 2.3. Sleep Duration

A sleep disorder questionnaire was used to evaluate sleep duration among adults aged 20–60 years old in the NHANES cycle of 2011–2016. The main question was “How much sleep do you usually get at night on weekdays or workdays?” The response documented was sleeping in hours. We categorized sleep into short sleep duration if sleep was less than 7 h and adequate sleep duration if sleep was 7 h and above [1].

### 2.4. Covariates

Covariates were selected based on previous knowledge [6,36] and were visualized in the direct acyclic graph (DAG), including age, gender (female and male), race, place of birth (born in the US), household education level, marital status (married, never married, and separated/widowed/divorced), family income to poverty ratio (PIR), food insecurity, waist circumference, BMI, physical activity, log_10_ serum cotinine (a biomarker of tobacco exposure), and log_10_ urine creatinine (Figure S2). In the obesity-specific analysis, BMI was categorized into general obesity (BMI ≥ 30 kg/m^2^) and no general obesity (BMI < 30 kg/m^2^), and waist circumference was categorized into abdominal obesity (waist circumference ≥ 102 cm in males and waist circumference ≥ 88 cm in females) and no abdominal obesity (waist circumference < 102 cm in males and waist circumference < 88 cm in females) [37].

### 2.5. Statistical Analysis

The data from the NHANES 2011–2012, 2013–2014, and 2015–2016 were analyzed and are presented. We present the descriptive statistics of the study population. The differences between the variables were summarized by the mean and standard deviation (SD) for continuous variables and count (n) and percentage (%) for categorical variables. The *t*-test was used for all continuous variables and the χ^2^ test was used for categorical variables. The descriptive statistics of all 17 PCPPCs were right-skewed. We transformed the log_10_ to all 17 PCPPCs to improve the normality. Pearson’s correlation coefficient was used to evaluate the correlation among the 17 PCPPCs, and the *p* < 0.05, 0.01, and 0.001 were set to be statistically significant.

#### 2.5.1. Logistic Regression

We conducted logistic regression to evaluate the gender- and obesity-specific effects of a single exposure to PCPPCs on short sleep duration among adults. The odds ratio (OR) and 95% confidence interval (CI) for each PCPPC were estimated. The models were adjusted for age, gender, race, country of birth (born in the US), guardian education, marital status, waist circumference, BMI, family income to poverty ratio (PIR), food insecurity, physical activity, log_10_ serum cotinine, and log_10_ urine creatinine, depending on the subgroup analysis. The *p* < 0.05 was set to be statistically significant. Since, logistic regression cannot account for the mixture effect, highly correlated chemicals, and nonlinear interactions between chemicals [37], weighted quantile sum (WQS) regression and Bayesian kernel machine regression (BKMR) were applied.

#### 2.5.2. Weighted Quantile Sum Regression

Weighted quantile sum (WQS) regression is a method used to evaluate the effect of mixed exposure on the outcomes and assign the burdened weight of each chemical in the mixture [38,39]. The model assigns quartiles of all chemicals and estimates weight for the WQS index to represent the overall effect of the mixture on the outcomes.

We randomly split the data into a training set of 40% (*n* = 1205) and validation set of 60% (*n* = 1807). We bootstrapped the training set 10,000 times to obtain the estimated weight of each PCPPC. To test the significance of each estimated weight in each bootstrap, we set a *p* < 0.05. The estimated weight was averaged to obtain empirical weights. The empirical weights were used to estimate a WQS index in the validation data that expresses the joint effect of the mixture of 17 PCPPCs on short sleep duration. We also set a value > 0.05 to determine the importance of each PCPPC contributing to the WQS index. The effect of the mixture of PCPPCs on short sleep duration was presented by gender and obesity status. We constructed three models for the adjustment of the covariates. Model 1: Unadjusted. Model 2: Adjusted for age, gender, race, education, marital status, body mass index (BMI), and waist circumference. Model 3: Adjusted for age, gender, race, education, marital status, BMI, waist circumference, family income to poverty ratio, food insecurity, physical activity, log_10_ cotinine, and log_10_ creatinine. We used the gWQS package (version 3.0.5) in R for the analysis.

#### 2.5.3. Bayesian Kernel Machine Regression

BKMR is a supervised non-parametric approach that assesses the effect of the mixture on the outcome. It uses a kernel function to stipulate the unidentified exposure–response association, permits nonlinear and interaction relations between chemicals, and incorporates the covariates [40]. In the present study, we implemented a hierarchical variable selection method by allocating the PCPPCs based on their sources and correlations as follows: group 1 comprises phenols (BZP, BPA, 2, 5-DCP, and 2, 4-DCP), group 2 comprises parabens (MeP and PrP), and group 3 comprises of phthalates (MCNP, MCOP, MECPP, MnBP, MCPP, MEP, MEHHP, MiBP, MEOHP, and MBzP). The posterior inclusion probability (PIP) at the threshold value of 0.5 was regarded to be significant. The model was fitted with the Markov Chain Monte Carlo (MCMC) algorithm for 10,000 iterations. We assessed the univariates exposure–outcome function with a 95% CI for PCPPCs with short sleep duration when setting the other PCPPCs at their median level. We then evaluated the pairwise interactions among chemicals in the mixture of PCPPCs on short sleep duration by the exposure–outcome function of a single exposure for the second exposure in the mixture of PCPPCs fixed at the 10th, 50th, and 90th percentiles with all remaining PCPPCs fixed at their median value. We assessed the joint effect of the mixtures of the PCPPCs on short sleep duration by comparing the difference when all of the PCPPCs were fixed at different percentiles (25th, 30th, 35th, 40th, 45th, 50th, 55th, 60th, 65th, 70th, or 75th) with their 50th percentile (median). All analyses were performed stratified by gender and obesity status. We used the bkmr package version 0.2.2 in R for analyses.

### 2.6. Sensitivity Analysis

To evaluate the robustness of our findings, we conducted a sensitivity analysis by repeating the analysis after including 56 underweight participants who were excluded from the primary analysis.

## 3. Results

### 3.1. Baseline Characteristics of the Study Population

The overall population of 3012 adults aged 20–60 years were included in this study. The baseline characteristics of the study population are presented in Table 1. The prevalence of short sleep duration was 37.1%. The mean ± SD ages of adults with short sleep and adequate sleep duration were (40.44 ± 11.65) and (39.26 ± 11.85) years, respectively. Gender, age, race, education level, marital status, waist circumference, BMI, general obesity, physical activity, cotinine, and creatinine were significant differences between short sleep and adequate sleep duration among adults.

### 3.2. Distributions of Urinary PCPPCs

The detection rates of the PCPPCs included in our study ranged from 72.7 to 99.7%. MEP had the highest detection rate (99.7%) and TCS had the lowest detection rate (72.7%). MeP had the highest GM (51.35) and 2, 4-DCP had the lowest GM (0.74) (Table S1). The Pearson correlations among all of the 17 PCPPCs were statistically significant at *p* < 0.001, with exceptional values assigned to TCS and 2, 5-DCP (r = 0.05), which were correlated at *p* < 0.01, and BZP and 2, 5-DCP (r = 0.02) were not correlated. The PCPPCs with the highest and lowest correlations were between MEHHP and MEOHP (r = 0.97), as well as BZP and 2, 5-DCP (r = 0.02), respectively (Figure S3).

### 3.3. Association between PCPPC Exposures and Short Sleep Duration by Gender and Obesity-Specific Status: Logistic Regression Findings

Table 2 shows the association between PCPPC exposures and short sleep duration by gender and obesity. The adjusted logistic regression showed that TCS (OR = 1.18, 95% CI: 1.04, 1.35; *p*-value = 0.012), MiBP (OR = 1.40, 95% CI: 1.02, 1.94; *p*-value = 0.039), and MBzP (OR = 1.56, 95% CI: 1.20, 2.04; *p*-value = 0.001) were significantly positively associated with short sleep duration in females only. BPA, MCNP, MCOP, MECPP, MCPP, MEHHP, and MEOHP were significantly positively associated with short sleep duration in both females and males.

Table 3 shows the association between PCPPC exposures and short sleep duration by obesity status. In general obesity, the adjusted logistic regression showed that MBzP (OR = 1.44, 95% CI: 1.09, 1.92, *p*-value = 0.011) was significantly positively associated with short sleep duration among participants with general obesity, while BPA (OR = 1.45, 95% CI: 1.12, 1.89; *p*-value = 0.005) and TCS (OR = 1.15, 95% CI: 1.02, 1.30; *p*-value = 0.022) were significantly positively associated with short sleep duration in participants with no general obesity. On the other hand, MCNP, MCOP, MECPP, MCPP, MEHHP, and MEOHP were significantly positively associated with short sleep duration in participants with both general obesity and no general obesity. In abdominal obesity, the adjusted logistic regression showed that MiBP (OR = 1.42, 95% CI: 1.05, 1.93; *p*-value = 0.022) and MBzP (OR = 1.41, 95% CI: 1.11, 1.80; *p*-value = 0.005) were significantly positively associated with short sleep duration in the participants with abdominal obesity, while BPA, MCNP, MCOP, MECPP, MCPP, MEHHP, and MEOHP were significantly positively associated with short sleep duration in participants with both abdominal obesity and no abdominal obesity.

### 3.4. Association between PCPPC Mixture and Short Sleep Duration by Gender- and Obesity-Specific Status: WQS Regression Findings

**Figure 1:**
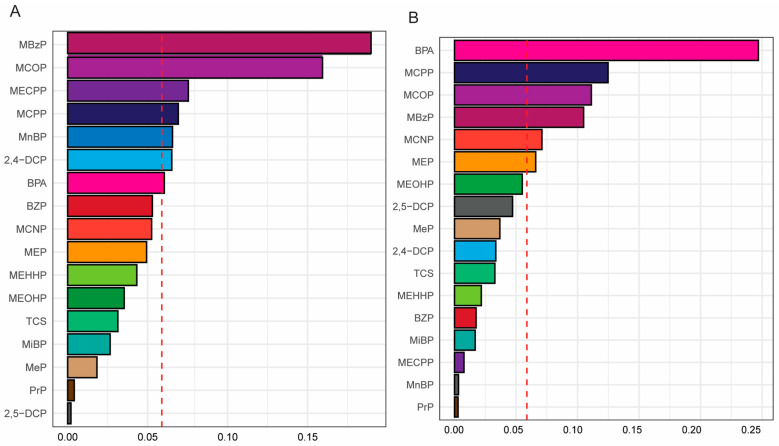
Weighted quantile sum (WQS) regression model index weight for a mixture of personal care product and plasticizing chemicals (PCPPCs) with short sleep duration among adults by gender-specific status. (**A**) Female. (**B**) Male. The red line indicates the cutoff point to discriminate the relative importance of the chemical. The model was adjusted for age, race, education, marital status, body mass index (BMI) [not adjusted for general obesity and no general obesity], waist circumference (not adjusted for abdominal obesity and no abdominal obesity), family income to poverty ratio (PIR), food insecurity, physical activity, log_10_ cotinine, and log_10_ creatinine.

**Figure 2:**
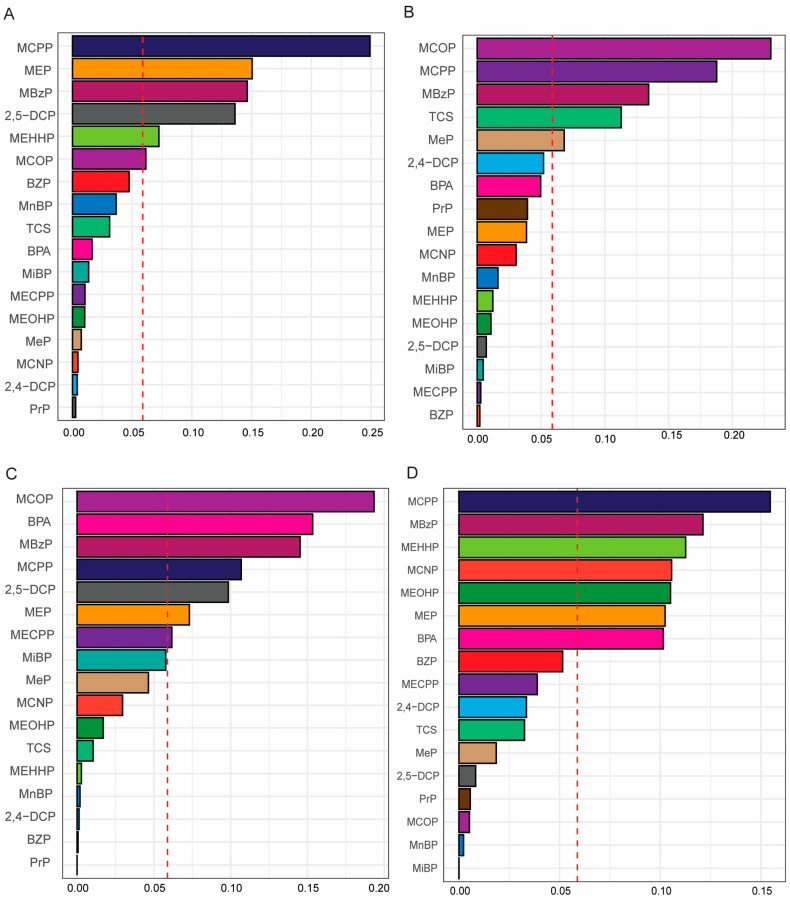
Weighted quantile sum (WQS) regression model index weight for a mixture of personal care product and plasticizing chemicals (PCPPCs) with short sleep duration among adults by obesity-specific status. (**A**) General obesity. (**B**) No general obesity. (**C**) Abdominal obesity. (**D**) No abdominal obesity. The red line indicates the cutoff point to discriminate the relative importance of the chemical. The model was adjusted for age, gender, race, education, marital status, body mass index (BMI) [not adjusted for general obesity and no general obesity], waist circumference (not adjusted for abdominal obesity and no abdominal obesity), family income to poverty ratio (PIR), food insecurity, physical activity, log_10_ cotinine, and log_10_ creatinine.

Table 4 shows the association between the WQS indices and short sleep duration by gender- and obesity-specific status. The WQS indices of the mixture of 17 PCPPCs were significantly positively associated with short sleep duration in both females (Model 3: OR = 1.55, 95% CI: 1.13, 2.11; *p*-value = 0.006) and males (Model 3: OR = 1.38, 95% CI: 1.03, 1.83; *p*-value = 0.029). For females, MBzP (weight = 0.19), MCOP (weight = 0.16), MECPP (weight = 0.08), MCPP, MnBP, and 2, 4-DCP (weight = 0.07), and BPA (weight = 0.06) contributed significantly to the WQS index for short sleep duration as they had a weight > 0.05, while, in males, BPA (weight = 0.25), MCPP (weight = 0.13), MCOP (weight = 0.11), MBzP (weight = 0.11), MCNP and MEP (weight = 0.07), and MEOHP (weight = 0.06) contributed significantly to the WQS index for short sleep duration (Figure 1 and Table S2). These results demonstrate that BPA, MCOP, and MCPP were of relative importance to short sleep duration in both female and male adults, while MBzP was of relative importance in females only, and MEOHP in males only, which was consistent with the findings in the logistic regression model. In the obesity-specific analysis, the WQS indices of the mixture of PCPPCs were positively significantly associated with short sleep duration among participants with general obesity (Model 3: OR = 1.40, 95% CI: 1.02, 1.92; *p*-value = 0.037), no general obesity (Model 3: OR = 1.36, 95% CI: 1.06, 1.75; *p*-value = 0.015), and abdominal obesity (Model 3: OR = 1.76, 95% CI: 1.33, 2.34; *p*-value < 0.001), but not those with no abdominal obesity (Model 3: OR = 1.30, 95% CI: 0.95, 1.78; *p*-value = 0.106) (Table 4). Meanwhile, MCPP (weight = 0.25), MEP (weight = 0.15), MBzP (weight = 0.15), 2, 5-DCP (weight = 0.14), MEHHP (weight = 0.07), and MCOP (weight = 0.06) contributed significantly to the WQS index for sleep duration in general obesity; MCOP (weight = 0.23), MCPP (weight = 0.18), MBzP (weight = 0.13), TCS (weight = 0.11), and MeP (weight = 0.07) had the highest contribution to the WQS index for short sleep in participants with no general obesity; and MCOP (weight = 0.19), BPA (weight = 0.15), MBzP (weight = 0.15), MCPP (weight = 0.11), 2, 5-DCP (weight = 0.10), MEP (weight = 0.07), and MECPP and MiBP (weight = 0.06) had the highest contribution to the WQS index for short sleep duration in abdominal obesity participants. In participants with no abdominal obesity, MCPP (weight = 0.16), MBzP (weight = 0.12), MEHHP (weight = 0.11), MCNP (weight = 0.11), MEOHP (weight = 0.11), MEP (weight = 0.10), BPA (weight = 0.10), and BZP (weight = 0.05) contributed to the WQS index for short sleep duration (Figure 2 and Table S2). These results demonstrate that MCOP, MCPP, and MBzP were relatively important exposures to short sleep duration in both general obesity and abdominal obesity, TCS and MEHHP in general obesity only, BPA and MiBP in abdominal obesity only, MCPP in both participants with neither general obesity nor abdominal obesity, MCOP and TCS in no general obesity only, and BPA, MCNP, MEHHP, and MEOHP in participants with no abdominal obesity only, similar to the findings in logistic regression model.

### 3.5. Association between PCPPC Mixture and Short Sleep Duration by Gender and Obesity: BKMR Findings

**Figure 3:**
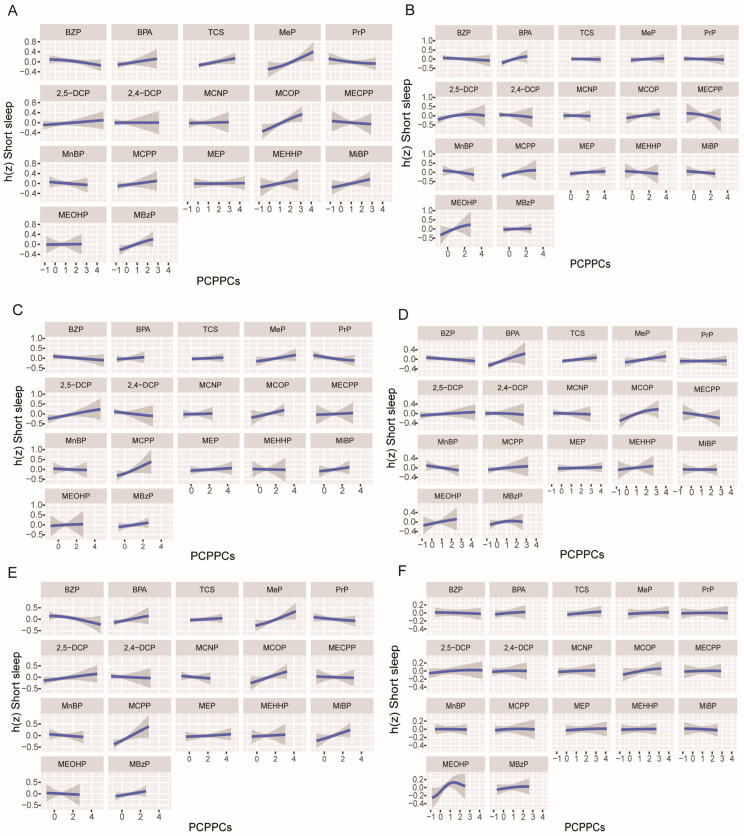
Univariate exposure–outcome function (95% credible interval) for personal care product and plasticizing chemicals (PCPPCs) with short sleep duration among adults by gender- and obesity-specific status. The function was estimated while fixing other PCPPCs at their median level. (**A**) Female. (**B**) Male. (**C**) General obesity. (**D**) No general obesity. (**E**) Abdominal obesity. (**F**) No abdominal obesity. The Bayesian kernel machine regression model was adjusted for age, gender (not adjusted for female and male), race, education, marital status, body mass index (BMI) [not adjusted for general obesity and no general obesity], waist circumference (not adjusted for abdominal obesity and no abdominal obesity), family income to poverty ratio (PIR), food insecurity, physical activity, log_10_ cotinine, and log_10_ creatinine.

PCPPCs were categorized into the following three groups: the phenol group, the paraben group, and the phthalate group. The group PIP results by gender and obesity status are presented in Table S3. Since PIP does not show the direction of the associations, we assessed the direction of single exposure to PCPPCs with short sleep duration when other PCPPCs were set at their median value (Figure 3). In both females and males, BPA, 2, 5-DCP, MCOP, and MCPP revealed a positive association with short sleep duration. TCS, MeP, MEHHP, MiBP, and MBzP revealed a positive association with short sleep duration only in females, while BZP and MECPP revealed a negative association. Furthermore, MEOHP showed a positive association in males, and MECPP and MnBP showed a negative association (Figure 3A,B). In comparison with the findings from other models, BPA, MCOP, and MCPP were positively associated with short sleep duration in both female and male adults, MBzP revealed a significant positive association in females only, and MEOHP in males only, which was consistent with the findings in the logistic regression and WQS regression models. On the other hand, TCS and MEHHP were positively associated with short sleep duration in females, which was consistent with the findings in the logistic regression model.

Moreover, in the obesity-specific analysis, MeP, 2, 5-DCP, MCOP, MCPP, MiBP, and MBzP were positively associated with short sleep duration, while BZP and PrP showed a negative association in both general and abdominal obesity, and BPA and MiBP revealed a positive association with short sleep duration in abdominal obesity only. In participants with no general obesity, BPA, MeP, MCOP, MCPP, MEHHP, and MEOHP were positively associated with short sleep duration, while BZP and MnBP revealed a negative association. In participants with no abdominal obesity, MEOHP was nonlinearly positively associated with short sleep duration (Figure 3C–F)

In comparison with the findings from other models, MCOP, MCPP, and MBzP were consistently positively associated with short sleep duration in both general obesity and abdominal obesity, and BPA and MiBP were positively associated with short sleep duration in abdominal obesity, which similar to the findings in the logistic regression and WQS regression models. Additionally, 2, 5-DCP was positively associated with short sleep duration in both general obesity and abdominal obesity, and had a higher weight in the WQS regression model. Moreover, BPA, MEHHP, and MEOHP revealed a positive association with short sleep duration in participants with no general obesity, which was consistent with the findings in the logistic regression model. MCPP was associated with short sleep duration in participants with no general obesity, which was consistent with the findings in the WQS regression and logistic regression models, while BPA and MEOHP revealed positive associations in participants with no abdominal obesity in all three models, and MCOP revealed a positive association, which was similar to the findings in the logistic regression model.

**Figure 4:**
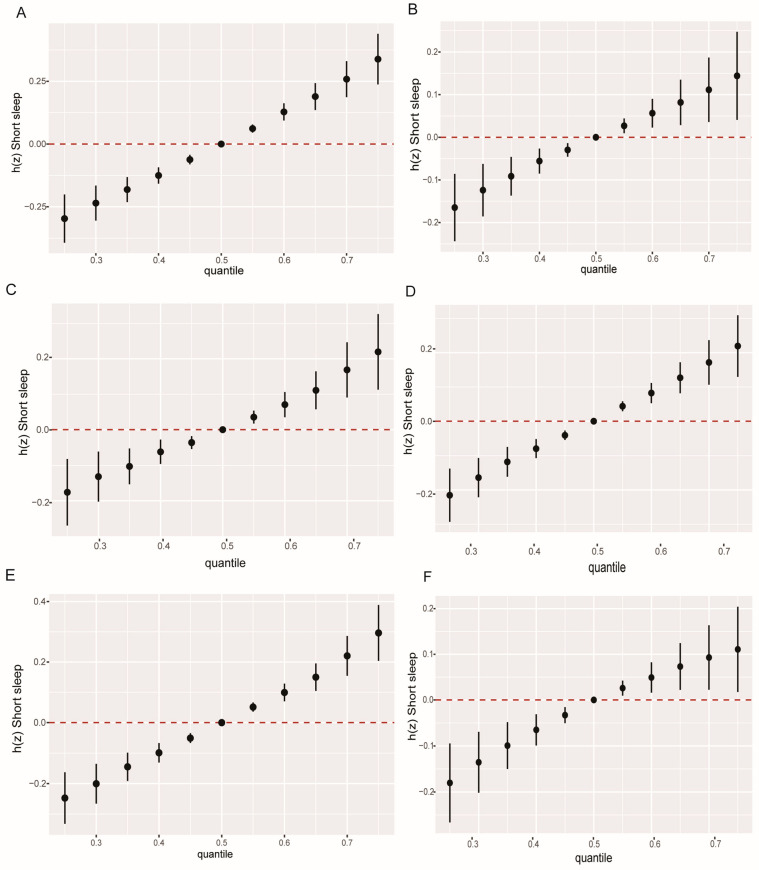
The mixture effect (95% confidence interval) of co-exposure to 17 personal care product and plasticizing chemicals (PCPPCs) on short sleep duration among adults by gender- and obesity-specific status. (**A**) Female. (**B**) Male. (**C**) General obesity. (**D**) No general obesity. (**E**) Abdominal obesity. (**F**) No abdominal obesity. The Bayesian kernel machine regression model was adjusted for age, gender (not adjusted for female and male), race, education, marital status, body mass index (BMI) [not adjusted for general obesity and no general obesity], waist circumference (not adjusted for abdominal obesity and no abdominal obesity), family income to poverty ratio (PIR), food insecurity, physical activity, log_10_ cotinine, and log_10_ creatinine.

Moreover, the mixture of 17 PCPPCs was consistently positively associated with short sleep duration irrespective of gender- and obesity-specific differences (Figure 4), which was similar to the findings in the WQS regression model, except for those with no abdominal obesity, in which no statistically significant association was observed in the WQS regression model. In addition, we observed possibly significant interactions between chemicals in the mixture of PCPPCs; particularly, there were possible bi-interactions between MBzP and each of MCOP, MeP, MiBP, MEHHP, MCPP, BPA, and 2,5-DCP; MCOP and each of MiBP, MiBP, MeP, MEHHP, MCPP, BPA, and 2, 5-DCP; MeP and each of MiBP, MEHHP, MCPP, BPA, and 2, 5-DCP in females, and some of these bi-interactions were observed in participants with no general obesity and those with abdominal obesity (Figures S4–S9).

### 3.6. Sensitivity Analysis Findings

Sensitivity analysis was conducted by including the underweight participants in the analysis who were excluded from the primary analysis, and the results in the logistic regression, WQS regression, and BKMR models were consistent with the primary analysis, suggesting the robustness of our findings (Tables S4–S6, and Figure S10).

## 4. Discussion

### 4.1. Principle Findings

In this national representative cross-sectional study, we investigated the gender- and obesity-specific association between co-exposure to personal care product and plasticizing chemicals (PCPPCs) and short sleep duration among adults. We found that BPA, MCOP, and MCPP were consistently associated with short sleep duration in both females and males, MBzP was associated with short sleep duration only in females, while MEOHP was associated only in males. Moreover, MCOP, MCPP, and MBzP were positively associated with short sleep duration in both general obesity and abdominal obesity, but BPA and MiBP were positively associated with short sleep duration in abdominal obesity only. On the other hand, BPA, MCOP, and MCPP were consistently associated with short sleep duration in participants with no general obesity and no abdominal obesity. These associations were consistent in the logistic regression, WQS regression, and BMKR models. Moreover, the mixture of 17 PCPPCs was consistently positively associated with short sleep duration irrespective of gender and obesity status in both the WQS regression and BKMR models, except for those with no abdominal obesity, in which no association was observed in the WQS regression. This indicates that single or combined chemicals can influence sleep duration regardless of demographic differences. In addition, there were possible interactions between PCPPCs in the mixture; particularly, bi-interactions were observed between MBzP and each of MCOP, MeP, MiBP, MEHHP, MCPP, BPA, and 2, 5-DCP; MCOP and each of MiBP, MiBP, MeP, MEHHP, MCPP, BPA, and 2, 5-DCP; MeP and each of MiBP, MEHHP, MCPP, BPA, and 2, 5-DCP in females, and some of these bi-interactions were observed in participants with no general obesity and those with abdominal obesity, leading to increased synergistic effects of the combined chemicals.

### 4.2. Comparison with Other Studies

Previous studies have explored the association between certain PCPPCs and sleep problems [6,7,8,9]. However, these studies did not differentiate their findings based on sex- or obesity-specific factors. Although a few studies have investigated the association between certain PCPPCs with sleep problems in females, the focus was only on phthalates [19,20,21], a kind of PCPPC, leaving the gap with respect to other types of PCPPCs such as phenols and parabens. Research evidence has reported significant gender differences in poor sleep quality among adults. In particular, females are more likely to have poor sleep quality compared to males [10,11]. Other studies have linked short sleep duration with obesity among females [12,13,14]. On the contrary, a study conducted in Japan reported an association between short sleep and general obesity among males, but not among females [41].

#### 4.2.1. Gender-Specific Differences

A previous study has reported a positive association between MECCP, MEP, MBzP, and MnBP with sleep problems in females. In addition, other studies have also linked phthalates with poor sleep quality in midlife women [19,20]. These results were in agreement with parts of our findings, as in the gender-specific analysis, where MCOP and MCPP were consistently positively associated with an increasing risk of short sleep duration in both female and male adults, while MBzP and MEHHP were associated with an increasing risk of short sleep duration in females, and MEOHP in males. These associations were consistent in at least two models. Furthermore, the mixture of 17 PCPPCs was consistently associated with an increasing risk of short sleep duration in both females and males in both the WQS regression and BKMR models, which was similar to the previous study [21]. The difference between previous studies and ours is that our study investigated several types of PCPPCs, including phthalates, phenols, and parabens, and the analyses were performed by gender- and obesity-specific associations, unlike these three studies [19,20,21], which focused only on phthalates and sleep problems in females. However, the previous studies and our own study provide substantiated evidence that PCPPCs may be associated with an increased risk of sleep problems.

Furthermore, BPA [6,9] and TCS [9] have been linked with inadequate sleep among adults. This association was observed in our study; however, the association between TCS and short sleep duration was observed in females, which was in agreement with the previous study [22], while BPA was consistently associated with an increasing risk of short sleep duration in both female and male adults. On the contrary, another study has found no association between exposure to BPA and TCS with sleeping disorders [8]. The inconsistent result could be caused by the different focus among our study and the previous study, as Shiue (2017) focused on sleeping disorders such as waking up at night, being unrested during the day, leg jerks, and leg cramps while sleeping [8], whereas our study focused on sleeping duration.

#### 4.2.2. Obesity-Specific Differences

In the obesity-specific analysis, our study showed that 2,5-DCP and MBzP were associated with an increased risk of short sleep duration in both general obesity and abdominal obesity, while MiBP was associated with an increased risk of short sleep duration in abdominal obesity only. These results indicate that associations between 2,5-DCP, MBzP, and MiBP with short sleep duration were influenced by general obesity and abdominal obesity. These associations were consistent in at least two models. Moreover, the mixture of 17 PCPPCs was consistently positively associated with short sleep duration regardless of obesity status in both WQS regression and BKMR models, except for those with no abdominal obesity, in which no association was observed in the WQS regression. These results suggest that, although the mixture of PCPPCs and most of the selected individual PCPPCs were independently associated with an increasing risk of short sleep duration, the effects of certain PCPPCs may be little modified by obesity status. We could not find data to explain the obesity-specific association between PCPPCs and short sleep duration, indicating that this area needs further investigation.

Previous studies have indirectly linked BPA to suboptimal sleep through its obesogenic features [16,17], as obesity has been associated with short sleep duration [18,42]. Kishman and colleagues have linked time in bed, total sleep time, sleep efficiency, and wake after sleep with body composition [15]. Another study documented MECPP, MMP, and MEHHP to be related to the increased risk of abdominal obesity in the general population. When the sex-specific analysis was performed, MEOHP, MMP, and MEHHP were associated with an increasing risk of abdominal obesity in females, while MBzP was negatively associated with abdominal obesity in females. No significant associations were observed in males [43]. The study further points out that there was a stronger positive relationship between phthalates and abdominal obesity than general obesity [43]. In our study, only 2,5-DCP and MBzP were associated with an increased risk of short sleep duration in both general obesity and abdominal obesity, while MiBP was associated with an increased risk of short sleep duration in abdominal obesity only. These results indicate that associations between 2,5-DCP, MBzP, and MiBP with short sleep duration were influenced by general obesity and abdominal obesity. The previous epidemiological study explored the association between phthalate metabolites and sleep duration in adolescents using NHANES data and suggested that phthalate and some of its metabolites including DEHP (MEOHP and MEHHP), MCNP, and MCOP were positively associated with short sleep duration [7]. Incongruent to our findings, MEOHP and MEHHP were associated with an increasing risk of short sleep duration in both participants with neither general obesity nor abdominal obesity, while MCNP was positively associated with short sleep duration in participants with no abdominal obesity only. Furthermore, in our study, we found that BPA, TCS, and MeP might independently be positively associated with short sleep duration among adults, which was in agreement with the previous studies [6,9]. The effects of BPA on thyroid autoimmunity [44] could cause this association. Similarly, we may speculate that TCS might be associated with short sleep duration due to its effect on thyroid hemostasis and autoimmunity [45]. In addition, phthalate and its metabolites may be associated with short sleep duration due to their ability to disturb neuronal circuitry and inhibit the development of a hormonal arbitrated mechanism that controls growth and sleep [8].

Apart from our study and that of Zhou et.al. [9], we did not find any evidence that revealed the association between parabens and sleeping duration, except a study by Shiue [8] that reported no association between parabens and sleeping disorders.

WQS regression and BKMR are recent statistical approaches that simultaneously assess the overall effects of the mixtures of environmental exposure, account for highly correlated chemicals, and also select important components in the mixture that contribute to the onset of the outcome. However, these methods may have also demerits. The WQS regression assumes the unidirectional assessment of the effect of the mixture on the outcome and it requires evaluation of the effect of the mixture in either a positive or negative direction but not both directions at once. Consequently, the interpretation of the results becomes ambiguous if both directions show significant results. BKMR can assess the pairwise interactions between chemicals in the mixture, but cannot produce PIPs of the interacted chemicals.

### 4.3. Research Implications

This study investigated the gender- and obesity-specific associations of co-exposure to PCPPCs and short sleep duration among adults using the traditional epidemiological approach to evaluate the individual effect and recent exposomic approaches to assess the co-exposure effect.

Overall, because of the endocrine-disrupting features of PCPPCs, it can be plausible that exposure to the mixture of PCPPCs or single exposure to certain PCPPCs, including phthalates, phenols, and parabens, might have effects on short sleep duration through disturbing the secretion of growth hormones which regulate the sleep/wake state [23]. PCPPCs might disrupt thyroid function and cause short sleep duration as well, since disruption of thyroid function has been linked to short sleep duration [24]. Further, PCPPCs can disrupt the melatonin pathway, the hormone that plays a key role in the sleep/wake cycle, leading to sleep disturbance. In addition, PCPPCs may be associated with short sleep duration through their ability to disrupt the central nervous system, as sleep is regulated by the central nervous system [25,26].

There is scarce data on the mechanism of gender differences in sleep; however, sex steroids have been reported to explain the difference in sleep patterns between females and males [26,46]. Testosterone in males and estrogens and progestins in females are the major sex steroid hormones believed to regulate sleep behaviors [46]. A cohort study in the US has documented that lower testosterone levels were associated with reduced sleep efficiency, increased nocturnal awakenings, and reduced time in slow-wave sleep in males [47]. In females, sleep problems have been linked with ovarian steroid variation, such as puberty, menstrual cycle, pregnancy, and menopausal transition [46]. On the other hand, PCPPCs have been linked to changes in sex steroid hormones such as a decrease in levels of testosterone, an increase in estrogens, and early menarche onset [48].

In the case of obesity, obesity has been linked with sleep through hormonal disruptions. The adipose tissues in obese persons can produce higher levels of leptin and ghrelin, which regulate appetite. Leptin and ghrelin imbalances can affect sleep patterns by disrupting the normal sleep/wake cycle, leading to increasing sleep disturbances [49].

### 4.4. Strengths and Limitations

Several strengths can be found in our study. Firstly, our study is the first to analyze the association between co-exposure to PCPPCs and short sleep duration in a nationally representative sample with specific attention to gender and obesity. In addition, we used three statistical approaches, including logistic regression, WQS regression, and BKMR. The use of the WQS regression and BKMR approaches allowed us to evaluate the joint toxicity of the mixture of 17 PCPPCs on short sleep duration, which could not be evaluated in the logistic regression model. Secondly, we have added important contributions to the literature about the potential toxicity of exposure to PCPPCs at single and mixture levels on short sleep duration among adults respective to gender and obesity status.

Our study has also some limitations. Firstly, the sleep duration assessment was subjective through a self-reported questionnaire that may lead to being either under-rated or over-rated. Secondly, PCPPCs are non-persistent chemicals and can be excreted from the body within 2 days; therefore, single-spot urine cannot reflect a real exposure. However, in this study, we assumed exposure to PCPPCs was quite stable among the participants due to the habitual use of personal care and consumer products or plastic materials containing these chemicals, as evidenced by their higher detection rates. Thirdly, our study is a cross-sectional design, so it does not provide an obvious causal relationship, and an epidemiological prospective cohort study is warranted to authenticate our results. Fourthly, when using NHANES data, it is recommended to use sample weights to obtain a representative population and unbiased findings. Each participant is given a sample weight that accounts for non-response. However, in the current study, we used unweighted data over weighted data because most of the covariates used in composing the sampling weight are also adjusted in the regression, which could lead to over-adjustment [38,39]. In addition, some of the statistical methods used in our study, such as weighted quantile sum (WQS) regression and Bayesian kernel machine regression (BKMR), cannot account for a complex survey weight. Several studies have used unweighted data over weighted data for similar reasons [37,40]. Fifthly our study reports the results for adults aged 20 to 60 years, and could not be generalized to children and adolescents. However, to our knowledge, this is the first study to investigate the gender- and obesity-specific association between co-exposure to PCPPCs, including phthalates, phenols, and parabens, with short sleep duration among adults.

## 5. Conclusions

This study showed that PCPPCs, such as phthalates, phenols, and parabens, were associated with an increasing risk of short sleep duration in adults both individually and as a mixture; however, gender- and obesity-specific differences may have little modification on the effect certain individual PCPPCs on short sleep duration. We suggest that reducing exposure to PCPPCs and emphasizing adequate sleep duration should be of public health concern to promote good health. Additionally, future prospective cohort studies should be conducted to authenticate the present findings.

## Figures and Tables

**Table 1:** Baseline characteristics of the study population (NHANES 2011–2016) based on sleep duration.

	Overall (N = 3012)	Short Sleep (N = 1118)	Adequate Sleep (N = 1894)	*p*-Value
Gender				0.042
Male	1451 (48.2)	566 (50.6)	885 (46.7)	
Female	1561 (51.8)	552 (49.4)	1009 (53.3)	
Age (Year) (mean (SD))	39.70 (11.79)	40.44 (11.65)	39.26 (11.85)	0.008
Race (%)				<0.001
Mexican	413 (13.7)	137 (12.3)	276 (14.6)	
Hispanic	329 (10.9)	126 (11.3)	203 (10.7)	
White	1068 (35.5)	355 (31.8)	713 (37.6)	
Black	690 (22.9)	330 (29.5)	360 (19.0)	
Asian	395 (13.1)	123 (11.0)	272 (14.4)	
Multi-racial	117 (3.9)	47 (4.2)	70 (3.7)	
Education (%)				0.002
Less than 9th grade	180 (6.0)	58 (5.2)	122 (6.4)	
9–11th grade	360 (12.0)	141 (12.6)	219 (11.6)	
High school	638 (21.2)	248 (22.2)	390 (20.6)	
College	981 (32.6)	396 (35.4)	585 (30.9)	
Graduate or above	853 (28.3)	275 (24.6)	578 (30.5)	
Marital status (%)				<0.001
Married	1837 (61.0)	646 (57.8)	1191 (62.9)	
Never married	760 (25.2)	281 (25.1)	479 (25.3)	
Separated, widowed, divorced	415 (13.8)	191 (17.1)	224 (11.8)	
PIR (%)				0.479
No poverty	2318 (77.0)	852 (76.2)	1466 (77.4)	
Poverty	694 (23.0)	266 (23.8)	428 (22.6)	
Food insecurity (%)				0.547
No	2034 (67.5)	747 (66.8)	1287 (68.0)	
Yes	978 (32.5)	371 (33.2)	607 (32.0)	
Waist circumference (mean (SD))	98.89 (17.08)	100.64 (17.49)	97.86 (16.75)	<0.001
Abdominal obesity (%)				0.06
No	1375 (45.7)	485 (43.4)	890 (47.0)	
Yes	1637 (54.3)	633 (56.6)	1004 (53.0)	
BMI (kg/m^2^) (mean (SD))	29.42 (7.13)	30.09 (7.41)	29.03 (6.93)	<0.001
General obesity (%)				0.001
No	1826 (60.6)	634 (56.7)	1192 (62.9)	
Yes	1186 (39.4)	484 (43.3)	702 (37.1)	
Physical activity (%)				<0.001
Yes	1168 (38.8)	480 (42.9)	688 (36.3)	
No	1844 (61.2)	638 (57.1)	1206 (63.7)	
Cotinine (mean (SD))	61.67 (130.71)	77.38 (148.56)	52.40 (117.99)	<0.001
Creatinine (mean (SD))	131.66 (86.37)	139.31 (88.89)	127.14 (84.55)	<0.001

Note: BMI, body mass index; SD, standard deviation; PIR, family income to poverty ratio. The *p*-values were obtained from the Chi-square (χ^2^) test for categorical variables and a *t*-test for continuous variables.

**Table 2:** Association between PCPPCs and short sleep duration by gender.

PCPPCs	Female (N = 1561)		Male (N = 1451)	
	OR (95% CI)	*p*-Value	OR (95% CI)	*p*-Value
Phenols				
BZP	0.98 (0.87, 1.10)	0.734	1.04 (0.90, 1.20)	0.631
BPA	1.39 (1.04, 1.84)	0.024	1.47 (1.11, 1.94)	0.007
TCS	1.18 (1.04, 1.35)	0.012	1.02 (0.90, 1.17)	0.728
2, 5-DCP	1.02 (0.90, 1.16)	0.724	1.09 (0.96, 1.25)	0.185
2, 4-DCP	1.12 (0.92, 1.36)	0.246	1.04 (0.85, 1.28)	0.679
Parabens				
MeP	1.17 (0.99, 1.38)	0.073	1.02 (0.87, 1.19)	0.834
PrP	1.03 (0.91, 1.17)	0.650	0.98 (0.86, 1.11)	0.748
Phthalate metabolites				
MCNP	1.56 (1.18, 2.06)	0.002	1.40 (1.07, 1.84)	0.015
MCOP	1.59 (1.30, 1.94)	<0.001	1.37 (1.13, 1.67)	0.001
MECPP	1.54 (1.14, 2.08)	0.005	1.38 (1.02, 1.87)	0.034
MnBP	1.29 (0.96, 1.73)	0.087	1.16 (0.86, 1.57)	0.323
MCPP	1.62 (1.31, 2.01)	<0.001	1.44 (1.17, 1.77)	0.001
MEP	1.01 (0.84, 1.22)	0.903	1.12 (0.93, 1.35)	0.218
MEHHP	1.59 (1.20, 2.13)	0.002	1.34 (1.00, 1.78)	0.048
MiBP	1.40 (1.02, 1.94)	0.039	1.07 (0.79, 1.46)	0.662
MEOHP	1.57 (1.17, 2.13)	0.003	1.50 (1.11, 2.03)	0.009
MBzP	1.56 (1.20, 2.04)	0.001	1.17 (0.91, 1.51)	0.207

PCPPCs, personal care product and plasticizing chemicals; OR, odds ratio; CI, confidence interval. *p*-value < 0.05. Multivariable logistic regression was conducted and adjusted odds ratios were estimated. The model was adjusted for age, race, education, marital status, body mass index (BMI), waist circumference, family income to poverty ratio (PIR), food insecurity, physical activity, country of birth (born in the US), log_10_ cotinine, and log_10_ creatinine.

**Table 3:** Association between PCPPCs and short sleep duration by obesity status.

PCPPCs	General Obesity ^a^ (N = 1186)		No General Obesity ^a^ (N = 1826)		Abdominal Obesity ^b^ (N = 1637)		No Abdominal Obesity ^b^ (N = 1375)	
	OR (95% CI)	*p*-Value	OR (95% CI)	*p*-Value	OR (95% CI)	*p*-Value	OR (95% CI)	*p*-Value
Phenols								
BZP	1.00 (0.86, 1.15)	0.989	0.99 (0.88, 1.11)	0.833	0.98 (0.87, 1.10)	0.722	0.99 (0.86, 1.13)	0.851
BPA	1.33 (0.97, 1.80)	0.072	1.45 (1.12, 1.89)	0.005	1.44 (1.11, 1.86)	0.006	1.37 (1.00, 1.87)	0.047
TCS	1.05 (0.91, 1.22)	0.493	1.15 (1.02, 1.30)	0.022	1.09 (0.96, 1.23)	0.206	1.12 (0.98, 1.29)	0.090
2, 5-DCP	1.08 (0.94, 1.24)	0.273	1.03 (0.91, 1.16)	0.682	1.06 (0.94, 1.19)	0.341	1.04 (0.90, 1.20)	0.612
2, 4-DCP	1.06 (0.86, 1.31)	0.584	1.09 (0.91, 1.32)	0.353	1.08 (0.91, 1.30)	0.378	1.07 (0.85, 1.34)	0.555
Parabens								
MeP	1.01 (0.85, 1.20)	0.883	1.06 (0.92, 1.22)	0.402	1.11 (0.95, 1.28)	0.180	0.98 (0.84, 1.14)	0.751
PrP	0.92 (0.81, 1.06)	0.259	1.02 (0.92, 1.13)	0.741	1.00 (0.89, 1.12)	0.993	0.96 (0.85, 1.08)	0.483
Phthalate metabolites								
MCNP	1.65 (1.20, 2.26)	0.002	1.32 (1.03, 1.69)	0.027	1.56 (1.19, 2.04)	0.001	1.38 (1.05, 1.83)	0.023
MCOP	1.59 (1.26, 2.00)	<0.001	1.38 (1.16, 1.64)	<0.001	1.62 (1.34, 1.97)	<0.001	1.31 (1.07, 1.61)	0.008
MECPP	1.48 (1.06, 2.08)	0.020	1.38 (1.05, 1.81)	0.023	1.50 (1.12, 2.01)	0.006	1.41 (1.04, 1.93)	0.029
MnBP	1.38 (1.00, 1.91)	0.054	1.06 (0.81, 1.37)	0.677	1.29 (0.98, 1.70)	0.065	1.08 (0.80, 1.46)	0.623
MCPP	1.76 (1.37, 2.27)	<0.001	1.39 (1.15, 1.67)	0.001	1.83 (1.48, 2.27)	<0.001	1.26 (1.03, 1.56)	0.026
MEP	1.05 (0.86, 1.29)	0.610	1.05 (0.87, 1.24)	0.670	1.07 (0.90, 1.28)	0.429	1.05 (0.86, 1.29)	0.618
MEHHP	1.41 (1.02, 1.94)	0.036	1.46 (1.12, 1.89)	0.005	1.46 (1.11, 1.93)	0.008	1.48 (1.10, 1.99)	0.010
MiBP	1.32 (0.93, 1.87)	0.119	1.09 (0.83, 1.45)	0.533	1.42 (1.05, 1.93)	0.022	0.98 (0.71, 1.34)	0.895
MEOHP	1.48 (1.06, 2.07)	0.021	1.49 (1.13, 1.95)	0.004	1.47 (1.10, 1.96)	0.009	1.60 (1.17, 2.18)	0.003
MBzP	1.44 (1.09, 1.92)	0.011	1.22 (0.97, 1.54)	0.089	1.41 (1.11, 1.80)	0.005	1.22 (0.94, 1.60)	0.137

PCPPCs, personal care product and plasticizing chemicals; OR, odds ratio; CI, confidence interval. *p*-value < 0.05. Multivariable logistic regression was conducted and adjusted odds ratios were estimated. The model was adjusted for age, gender, race, education, marital status, body mass index (BMI), waist circumference, family income to poverty ratio (PIR), food insecurity, physical activity, country of born (born in the U.S.), log_10_ cotinine, and log_10_ creatinine. ^a^ BMI was not adjusted in the model. ^b^ Waist circumference was not adjusted in the model.

**Table 4:** Association between the WQS index and short sleep duration stratified by gender- and obesity-specific status.

Variables	Model	OR (95%CI)	*p*-Value
Female ^a^			
	Model 1	1.44 (1.20, 1.72)	<0.001
	Model 2	1.24 (1.02, 1.50)	0.031
	Model 3	1.55 (1.13, 2.11)	0.006
Male ^a^			
	Model 1	1.27 (1.06, 1.53)	0.011
	Model 2	1.24 (1.03, 1.50)	0.026
	Model 3	1.38 (1.03, 1.83)	0.029
General obesity ^b^			
	Model 1	1.36 (1.10, 1.68)	0.005
	Model 2	1.20 (0.96, 1.52)	0.111
	Model 3	1.40 (1.02, 1.92)	0.037
No general obesity ^b^			
	Model 1	1.28 (1.09, 1.52)	0.003
	Model 2	1.18 (1.00, 1.40)	0.047
	Model 3	1.36 (1.06, 1.75)	0.015
Abdominal obesity ^c^			
	Model 1	1.53 (1.28, 1.83)	<0.001
	Model 2	1.40 (1.17, 1.69)	<0.001
	Model 3	1.76 (1.33, 2.34)	<0.001
No abdominal obesity ^c^			
	Model 1	1.06 (0.88, 1.27)	0.546
	Model 2	1.00 (0.82, 1.22)	0.983
	Model 3	1.30 (0.95, 1.78)	0.106

Note: OR, odds ratio; CI, confidence interval. OR estimates represent the odds ratios of short sleep when the weighted quantile sum (WQS) index was increased by 1 quartile. Model 1: Unadjusted. Model 2: Adjusted for age, gender, race, education, marital status, body mass index (BMI), and waist circumference. Model 3: Adjusted for age, gender, race, education, marital status, BMI, waist circumference, family income to poverty ratio (PIR), food insecurity, physical activity, log_10_ cotinine, and log_10_ creatinine. ^a^ Gender was not adjusted in the model. ^b^ BMI was not adjusted in the model. ^c^ Waist circumference was not adjusted in the model.

## Data Availability

The data presented in this study are openly available in the National Health and Nutrition Examination Survey repository at https://wwwn.cdc.gov/nchs/nhanes/continuousnhanes/default.aspx (accessed on 25 May 2023). Datasets analyzed in the current study are available from the corresponding author on reasonable request.

## Supplementary Materials

The following supporting information can be downloaded at: https://www.mdpi.com/article/10.3390/toxics12070503/s1, Figure S1: Flowchart of participants included in the final analysis; Figure S2. Direct acyclic graph (DAG) of selecting adjustment sets for estimating the total effect of PCPPCs on short sleep duration among adults; Figure S3. Pairwise Pearson correlations among urine concentrations of 17 PCPPCs in the population (N = 3012). *, *p* < 0.05; ** *p* < 0.01; ***, *p* < 0.001; Figure S4. Bivariate exposure–outcome function between personal care product and plasticizing chemicals (PCPPCs) fixed on the top and short sleep duration among adults while fixing the PCPPCs on the right at 10th, 50th, and 90th percentiles in females; Figure S5. Bivariate exposure–outcome function between personal care product and plasticizing chemicals (PCPPCs) fixed on the top and short sleep duration among adults while fixing the PCPPCs on the right at 10th, 50th, and 90th percentiles in males; Figure S6. Bivariate exposure–outcome function between personal care product and plasticizing chemicals (PCPPCs) fixed on the top and short sleep duration among adults while fixing the PCPPCs on the right at 10th, 50th, and 90th percentiles in general obesity; Figure S7. Bivariate exposure–outcome function between personal care product and plasticizing chemicals (PCPPCs) fixed on the top and short sleep duration among adults while fixing the PCPPCs on the right at 10th, 50th, and 90th percentiles in no general obesity; Figure S8. Bivariate exposure–outcome function between personal care product and plasticizing chemicals (PCPPCs) fixed on the top and short sleep duration among adults while fixing the PCPPCs on the right at 10th, 50th, and 90th percentiles in abdominal obesity; Figure S9. Bivariate exposure–outcome function between personal care product and plasticizing chemicals (PCPPCs) fixed on the top and short sleep duration among adults while fixing the PCPPCs on the right at 10th, 50th, and 90th percentiles in no abdominal obesity; Figure S10. The mixture effect (95% confidence interval) of co-exposure to 17 personal care product and plasticizing chemicals (PCPPCs) on short sleep duration among adults by gender- and obesity-specific status after including 56 underweight participants in the primary analysis. (A) Female. (B) Male. (C) General obesity. (D) No general obesity. (E) Abdominal obesity. (F) No abdominal obesity; Table S1: Distribution of the concentration of PCPPCs among adults (N = 3012); Table S2. Estimated WQS index weights in short sleep duration by gender- and obesity-specific status; Table S3. Bayesian kernel machine regression hierarchical posterior inclusion probabilities (PIPs) for group and conditional with short sleep duration among adults by gender- and obesity-specific status (N = 3012); Table S4. Logistic regression for the association between PCPPCs and short sleep duration by gender-specific status (N = 3068); Table S5. Logistic regression for the association between PCPPCs and short sleep duration by obesity-specific status (N = 3068); Table S6: Association between the WQS index and short sleep duration in a positive direction stratified by gender- and obesity-specific status (N = 3068).
