# Supplementary material for: Gender- and Obesity-Specific Association of Co-Exposure to Personal Care Product and Plasticizing Chemicals and Short Sleep Duration among Adults: Evidence from the National Health and Nutrition Examination Survey 2011–2016
Source: Toxics. 2024 Jul 11;12(7):503. doi: 10.3390/toxics12070503 (PMC11281163; doi:10.3390/toxics12070503)
Supplement: Supplementary file 1 [file toxics-12-00503-s001.zip › toxics-3077976-supplementary.pdf]

Supplementary Materials

Supplementary Tables

Table S1. Distribution of the concentration of PCPPCs among adults (N=3012).

Exposure biomarkers	LOD (ng/mL)	DR (%)	GM	Mean	Percentiles						
					Min	5th	25th	50th	75th	95th	Max
Phenols (ng/mL)											
BZP	0.40	96.75	21.13	305.92	0.28	0.80	5.28	16.80	70.75	1102.14	99886.70
BPA	0.20	94.95	1.33	2.95	0.14	0.20	0.60	1.30	2.70	8.10	792.00
TCS	1.70	72.68	9.32	80.56	1.2	1.20	1.63	5.50	31.73	458.36	3629.10
2,5-DCP	0.10	97.48	4.70	152.51	0.07	0.20	1.00	3.40	17.03	346.05	41600.00
2,4-DCP	0.10	92.07	0.74	4.62	0.07	0.10	0.30	0.70	1.50	10.45	1260.60
Parabens (ng/mL)											
MeP	1.00	98.87	51.35	217.23	0.71	2.90	13.40	51.50	195.60	923.45	15964.10
PrP	0.10	97.94	6.89	54.97	0.07	0.20	1.10	7.00	39.20	256.79	3915.00
Phthalates (ng/mL)											
MCNP	0.20	97.24	2.20	4.73	0.14	0.30	1.10	2.10	4.40	14.39	876.40
MCOP	0.30	99.67	15.22	46.34	0.21	1.60	5.50	14.00	42.13	199.66	1813.10
MECPP	0.40	99.60	10.54	19.65	0.14	1.80	5.60	10.90	20.40	56.94	1548.40
MnBP	0.40	96.45	8.90	18.83	0.28	0.80	4.50	10.30	20.13	49.98	1343.90
MCPP	0.40	84.53	1.88	7.59	0.14	0.28	0.70	1.70	4.30	20.54	1537.90
MEP	1.20	99.70	42.08	173.90	0.42	4.40	14.10	37.40	111.43	570.52	30321.00
MEHHP	0.40	98.90	6.77	13.37	0.14	1.00	3.50	7.20	13.50	39.40	1186.60
MiBP	0.80	95.88	7.80	14.82	0.14	1.00	3.90	8.55	16.40	45.09	627.00
MEOHP	0.20	99.14	4.27	8.11	0.14	0.60	2.20	4.60	8.70	22.75	528.70
MBzP	0.30	96.64	4.23	10.02	0.21	0.50	1.80	4.40	10.30	36.24	431.20

PCPPCs, personal care product and plasticizing chemicals; LOD, limit of detection; DR, detection rate, GM, geometric mean.

Table S2. Estimated WQS index weights in short sleep duration by gender and obesity-specific status.

Mixture of PCPPCs	Short sleep duration					
	**Females**	**Males**	**General Obesity**	**No general obesity**	**Abdominal obesity**	**No abdominal obesity**
	**WQS index weights**	**WQS index weights**	**WQS index weights**	**WQS index weights**	**WQS index weights**	**WQS index weights**
**Phenols**						
BZP	0.05	0.02	0.05	<0.01	<0.01	0.05
BPA	0.06	0.25	0.02	0.05	0.15	0.10
TCS	0.03	0.03	0.03	0.11	0.01	0.03
2,5-DCP	<0.01	0.05	0.14	0.01	0.10	0.01
2,4-DCP	0.07	0.03	<0.01	0.05	<0.01	0.03
**Parabens**						
MeP	0.02	0.04	0.01	0.07	0.05	0.02
PrP	<0.01	<0.01	<0.01	0.04	0.00	0.01
**Phthalates**						
MCNP	0.05	0.07	<0.01	0.03	0.03	0.11
MCOP	0.16	0.11	0.06	0.23	0.19	0.01
MECPP	0.08	0.01	0.01	<0.01	0.06	0.04
MnBP	0.07	<0.01	0.04	0.02	<0.01	<0.01
MCPP	0.07	0.13	0.25	0.19	0.11	0.16
MEP	0.05	0.07	0.15	0.04	0.07	0.10
MEHHP	0.04	0.02	0.07	0.01	<0.01	0.11
MiBP	0.03	0.02	0.01	<0.01	0.06	<0.01
MEOHP	0.04	0.06	0.01	0.01	0.02	0.11
MBzP	0.19	0.11	0.15	0.13	0.15	0.12

PCPPCs, personal care product and plasticizing chemicals; WQS, weighted quantile sum. Estimated WQS index weights of Table 3 model 3 in positive constrained

Table S3. Bayesian kernel machine regression hierarchical posterior inclusion probabilities (PIPs) for group and conditional with short sleep duration among adults by gender and obesity-specific status (N =3012).

PCPPCs	Group	Female		Male		General obesity		No general obesity		Abdominal obesity		No abdominal obesity	
		Group PIP	Cond PIP	Group PIP	Cond PIP	Group PIP	Cond PIP	Group PIP	Cond PIP	Group PIP	Cond PIP	Group PIP	Cond PIP
**Phenols**													
BZP	1	0.53	0.08	0.62	0.02	0.33	0.03	0.79	0.02	0.66	0.19	0.67	0.12
BPA	1	0.53	0.36	0.62	0.73	0.33	0.25	0.79	0.82	0.66	0.50	0.67	0.20
TCS	1	0.53	0.33	0.62	0.01	0.33	0.03	0.79	0.07	0.66	0.03	0.67	0.22
2,5-DCP	1	0.53	0.05	0.62	0.21	0.33	0.53	0.79	0.03	0.66	0.17	0.67	0.25
2,4-DCP	1	0.53	0.17	0.62	0.03	0.33	0.15	0.79	0.05	0.66	0.11	0.67	0.22
**Parabens**													
MeP	2	0.84	0.89	0.22	0.74	0.22	0.53	0.69	0.81	0.81	0.90	0.60	0.55
PrP	2	0.84	0.11	0.22	0.26	0.22	0.47	0.69	0.19	0.81	0.10	0.60	0.45
**Phthalates**													
MCNP	3	0.97	<0.01	0.79	0.04	0.99	0.02	0.92	0.03	1.00	0.00	0.91	0.05
MCOP	3	0.97	0.82	0.79	0.21	0.99	0.20	0.92	0.68	1.00	0.11	0.91	0.07
MECPP	3	0.97	<0.01	0.79	0.06	0.99	0.00	0.92	0.01	1.00	0.00	0.91	0.06
MnBP	3	0.97	<0.01	0.79	0.01	0.99	0.00	0.92	<0.01	1.00	0.00	0.91	0.02
MCPP	3	0.97	0.17	0.79	0.52	0.99	0.77	0.92	0.18	1.00	0.89	0.91	0.06
MEP	3	0.97	0.00	0.79	0.03	0.99	0.00	0.92	0.01	1.00	0.00	0.91	0.02
MEHHP	3	0.97	0.00	0.79	0.03	0.99	<0.01	0.92	0.02	1.00	0.00	0.91	0.06
MiBP	3	0.97	0.00	0.79	0.01	0.99	0.00	0.92	<0.01	1.00	0.00	0.91	0.01
MEOHP	3	0.97	0.00	0.79	0.09	0.99	0.01	0.92	0.06	1.00	0.00	0.91	0.64
MBzP	3	0.97	0.01	0.79	0.02	0.99	<0.01	0.92	<0.01	1.00	0.00	0.91	0.02

Table S4. Logistic regression for the association between PCPPCs and short sleep duration by gender-specific status (N=3068).

	Female (N = 1599)		Male (N =1469)	
	**OR (95% CI)**	***p*-Value**	**OR (95% CI)**	***p*-Value**
**Phenols**				
BZP	0.16 (0.05, 0.53)	0.763	1.03 (0.89, 1.19)	0.661
BPA	1.42 (1.07, 1.87)	0.015	1.50 (1.13, 1.98)	0.005
TCS	1.18 (1.04, 1.34)	0.013	1.03 (0.90, 1.18)	0.651
2,5-DCP	1.04 (0.92, 1.18)	0.551	1.11 (0.97, 1.27)	0.118
2,4-DCP	1.13 (0.93, 1.37)	0.209	1.07 (0.87, 1.31)	0.521
**Parabens**				
MeP	1.17 (0.99, 1.38)	0.061	1.01 (0.86, 1.180)	0.896
PrP	1.04 (0.91, 1.18)	0.571	0.97 (0.86, 1.10)	0.682
**Phthalates metabolites**				
MCNP	1.57 (1.19, 2.06)	0.001	1.41 (1.07, 1.85)	0.013
MCOP	1.60 (1.32, 1.96)	<0.001	1.36 (1.12, 1.66)	0.002
MECPP	1.57 (1.16, 2.13)	0.003	1.40 (1.04, 1.89)	0.027
MnBP	1.36 (1.02, 1.81)	0.038	1.20 (0.89,1.61)	0.226
MCPP	1.64 (1.32, 2.03)	<0.001	1.44 (1.18, 1.77)	0.366
MEP	1.01 (0.83, 1.22)	0.945	1.12 (0.93, 1.34)	0.238
MEHHP	1.63 (1.23, 2.17)	0.001	1.36 (1.02, 1.81)	0.035
MiBP	1.48 (1.08, 2.04)	0.016	1.08 (0.79, 1.46)	0.640
MEOHP	1.59 (1.18, 2.14)	0.002	1.53 (1.13, 2.06)	0.006
MBzP	1.52 (1.17, 1.98)	0.002	1.20 (0.94, 1.54)	0.149

PCPPCs; personal care product and plasticizing chemicals; OR, odds ratio; CI, confidence interval. *P*-value <0.05. Multivariable logistic regression was conducted and adjusted odds ratios were estimated. The model was adjusted for age, race, education, marital status, body mass index (BMI), waist circumference, family income to poverty ratio, food insecurity, physical activity, country of born (born in the U.S.), $\log_{10}$ cotinine, and $\log_{10}$ creatinine. N = 3068 indicates the sample size after including 56 underweight participants in the primary analysis.

Table S5. Logistic regression for the association between PCPPCs and short sleep duration by obesity-specific status (N=3068).

	General Obesity [a] (N=1186)		No general Obesity [a] (N =1882)		Abdominal Obesity [b] (N =1637)		No abdominal Obesity [b] (N= 1431)	
	**OR (95%CI)**	***p*- Value**	**OR (95%CI)**	***p*- Value**	**OR (95% CI)**	***p*- Value**	**OR (95%CI)**	***p*- Value**
**Phenols**								
BZP	1.00 (0.86, 1.15)	0.989	0.98 (0.88, 1.10)	0.779	0.98 (0.87, 1.10)	0.722	0.98 (0.86, 1.12)	0.761
BPA	1.33 (0.97, 1.80)	0.072	1.50 (1.16, 1.94)	0.002	1.44 (1.11, 1.86)	0.006	1.44 (1.06, 1.95)	0.019
TCS	1.05 (0.91, 1.22)	0.493	1.15 (1.02, 1.30)	0.019	1.09 (0.96, 1.23)	0.206	1.13 (0.98, 1.29)	0.085
2,5-DCP	1.08 (0.94, 1.24)	0.273	1.05 (0.93, 1.18)	0.419	1.06 (0.94, 1.19)	0.341	1.07 (0.93, 1.23)	0.357
2,4-DCP	1.06 (0.86, 1.31)	0.584	1.12 (0.93, 1.34)	0.246	1.08 (0.91, 1.30)	0.378	1.10 (0.88, 1.36)	0.411
**Parabens**								
MeP	1.01 (0.85, 1.20)	0.883	1.05 (0.92, 1.20)	0.477	1.11(0.95, 1.28)	0.180	0.96(0.83, 1.12)	0.643
PrP	0.92 (0.81, 1.06)	0.259	1.01 (0.91, 1.12)	0.823	1.00(0.89, 1.12)	0.993	0.95(0.84, 1.07)	0.415
**Phthalates metabolites**								
MCNP	1.65 (1.20, 2.26)	0.002	1.33 (1.04, 1.70)	0.021	1.56 (1.19, 2.04)	0.001	1.40 (1.06, 1.84)	0.017
MCOP	1.59 (1.26, 2.00)	<0.001	1.38 (1.16, 1.64)	<0.001	1.62(1.34, 1.97)	<0.001	1.31 (1.08, 1.60)	0.007
MECPP	1.48 (1.06, 2.08)	0.020	1.40 (1.07, 1.84)	0.014	1.50 (1.12, 2.01)	0.006	1.44 (1.06, 1.96)	0.019
MnBP	1.38 (1.00, 1.91)	0.054	1.13 (0.87, 1.46)	0.359	1.29 (0.98, 1.70)	0.065	1.17 (0.87, 1.57)	0.298
MCPP	1.76 (1.37, 2.27)	<0.001	1.40 (1.17, 1.68)	<0.001	1.83 (1.48, 2.27)	<0.001	1.28 (1.04, 1.57)	0.017
MEP	1.05 (0.86, 1.29)	0.610	1.03 (0.87, 1.22)	0.731	1.07 (0.90, 1.28)	0.429	1.04 (0.85, 1.27)	0.689
MEHHP	1.41 (1.02, 1.94)	0.036	1.50 (1.16, 1.95)	0.002	1.46 (1.11, 1.93)	0.008	1.53 (1.14, 2.05)	0.005
MiBP	1.32 (0.93, 1.87)	0.119	1.15 (0.87, 1.51)	0.333	1.42 (1.05, 1.93)	0.022	1.04 (0.76, 1.41)	0.816
MEOHP	1.48 (1.06, 2.07)	0.021	1.51 (1.15, 1.97)	0.003	1.47 (1.10, 1.96)	0.009	1.61 (1.19, 2.18)	0.002
MBzP	1.44 (1.09, 1.92)	0.011	1.22 (0.97, 1.53)	0.086	1.41 (1.11, 1.80)	0.005	1.22 (0.94, 1.59)	0.131

PCPPCs; personal care product and plasticizing chemicals; OR, odds ratio; CI, confidence interval. *P*-value <0.05. Multivariable logistic regression was conducted and adjusted odds ratios were estimated. The model was adjusted for age, gender, race, education, marital status, body mass index (BMI), waist circumference, family income to poverty ratio, food insecurity, physical activity, country of born (born in the U.S.), log cotinine, and log creatinine.

N =3068 indicates the sample size after including 56 underweight participants in the primary analysis.

[a] BMI was not adjusted in the model.

[b] Waist circumference was not adjusted in the model.

Table S6: Association between the WQS index and short sleep duration in a positive direction stratified by gender and obesity-specific (N =3068).

Variables	Model	OR (95%CI)	*p*-Value
Female [a]			
	Model 1	1.48 (1.23, 1.79)	<0.001
	Model 2	1.32 (1.09, 1.61)	0.005
	Model 3	1.53 (1.13, 2.07)	0.006
Male [a]			
	Model 1	1.44 (1.20, 1.73)	<0.001
	Model 2	1.35 (1.12, 1.63)	0.001
	Model 3	1.51 (1.13, 2.02)	0.006
General obesity [b]			
	Model 1	1.36 (1.10, 1.68)	0.005
	Model 2	1.20 (0.96, 1.52)	
	Model 3	1.40 (1.02, 1.92)	0.037
No general obesity [b]			
	Model 1	1.39 (1.18, 1.63)	<0.001
	Model 2	1.30 (1.10, 1.54)	0.002
	Model 3	1.72 (1.31, 2.27)	<0.001
Abdominal obesity [c]			
	Model 1	1.53 (1.28, 1.83)	<0.001
	Model 2	1.40 (1.17, 1.69)	<0.001
	Model 3	1.762 (1.33, 2.34)	<0.001
No abdominal obesity [c]			
	Model 1	1.24 (1.02, 1.50)	0.029
	Model 2	1.173 (0.96,1.43)	0.119
	Model 3	1.78 (1.28, 2.48)	0.002

Note: OR, odds ratio; CI, confidence interval. OR estimates represent the odds ratios of short sleep when the weighted quantile sum (WQS) index was increased by 1 quartile. Model 1: Unadjusted. Model 2: Adjusted for age, gender, race, education, marital status, body mass index (BMI), and waist circumference. Model 3: Adjusted for age, gender, race, education, marital status, BMI, waist circumference, family income to poverty ratio, food insecurity, physical activity, log cotinine, and log creatinine.

N=3068 indicates the sample size after including 56 underweight participants in the primary analysis.

[a] Gender was not adjusted in the model.

[b] BMI was not adjusted in the model.

[c] Waist circumference was not adjusted in the model.

Supplementary Figures

Figure S1. Flowchart of participants included in the final analysis.

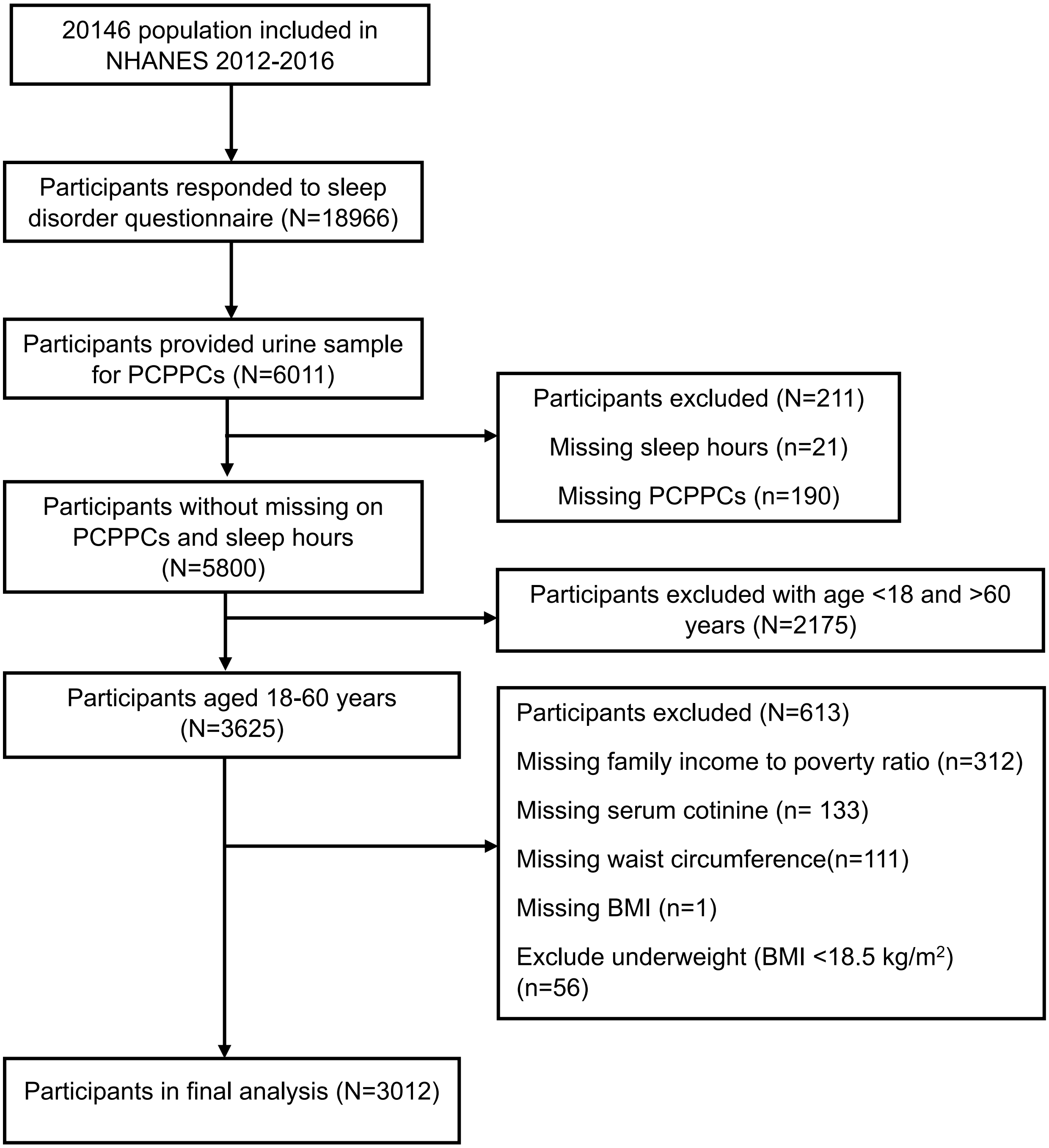

Figure S2. Direct acyclic graph (DAG) of selecting adjustment sets for estimating the total effect of PCPPCs on short sleep duration among adults.

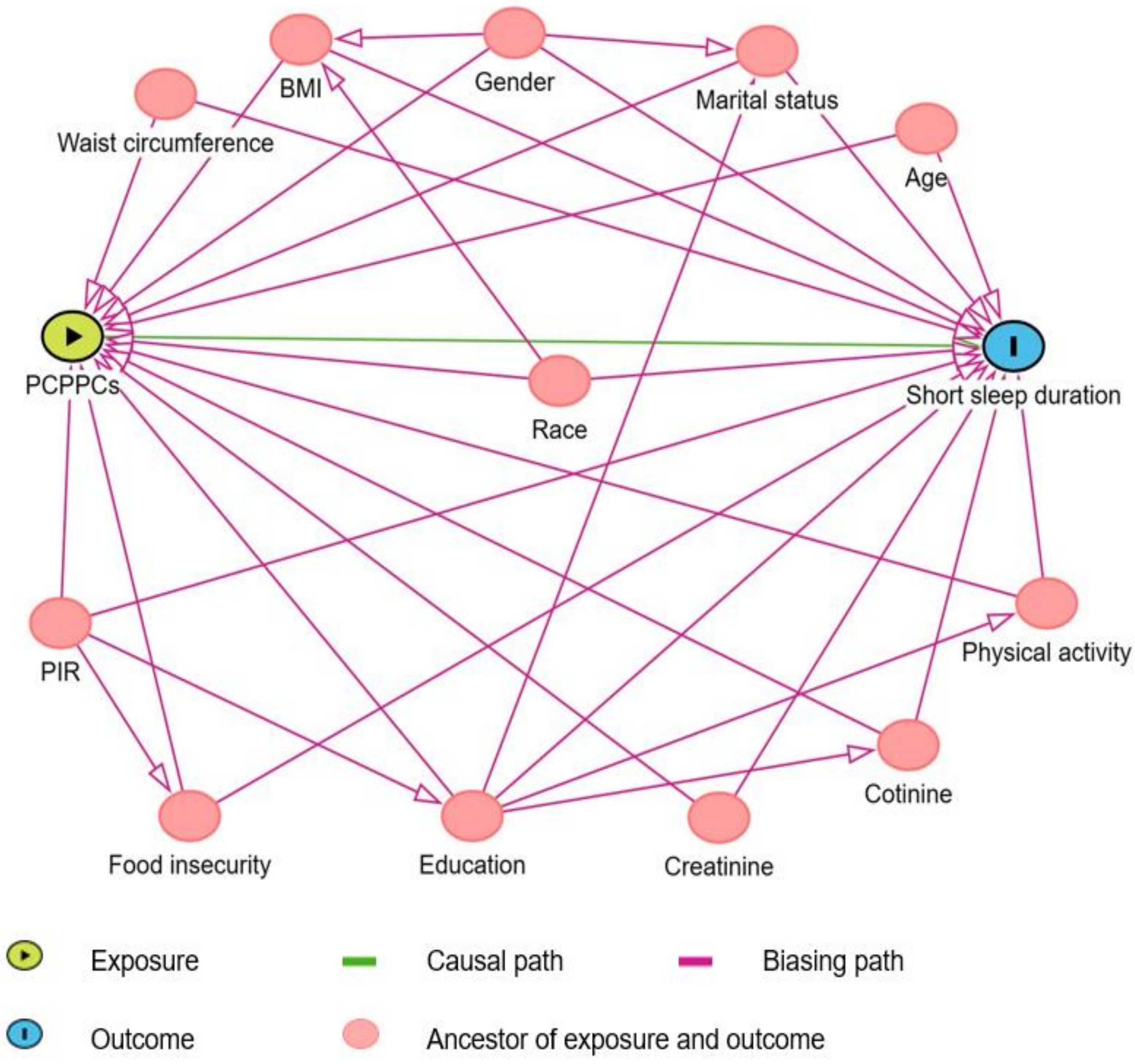

Figure S3. Pairwise Pearson correlations among urine concentrations of 17 PCPPCs in the population (N=3012). *, *p* <0.05; ** *p*<0.01; ***, *p*< 0.001.

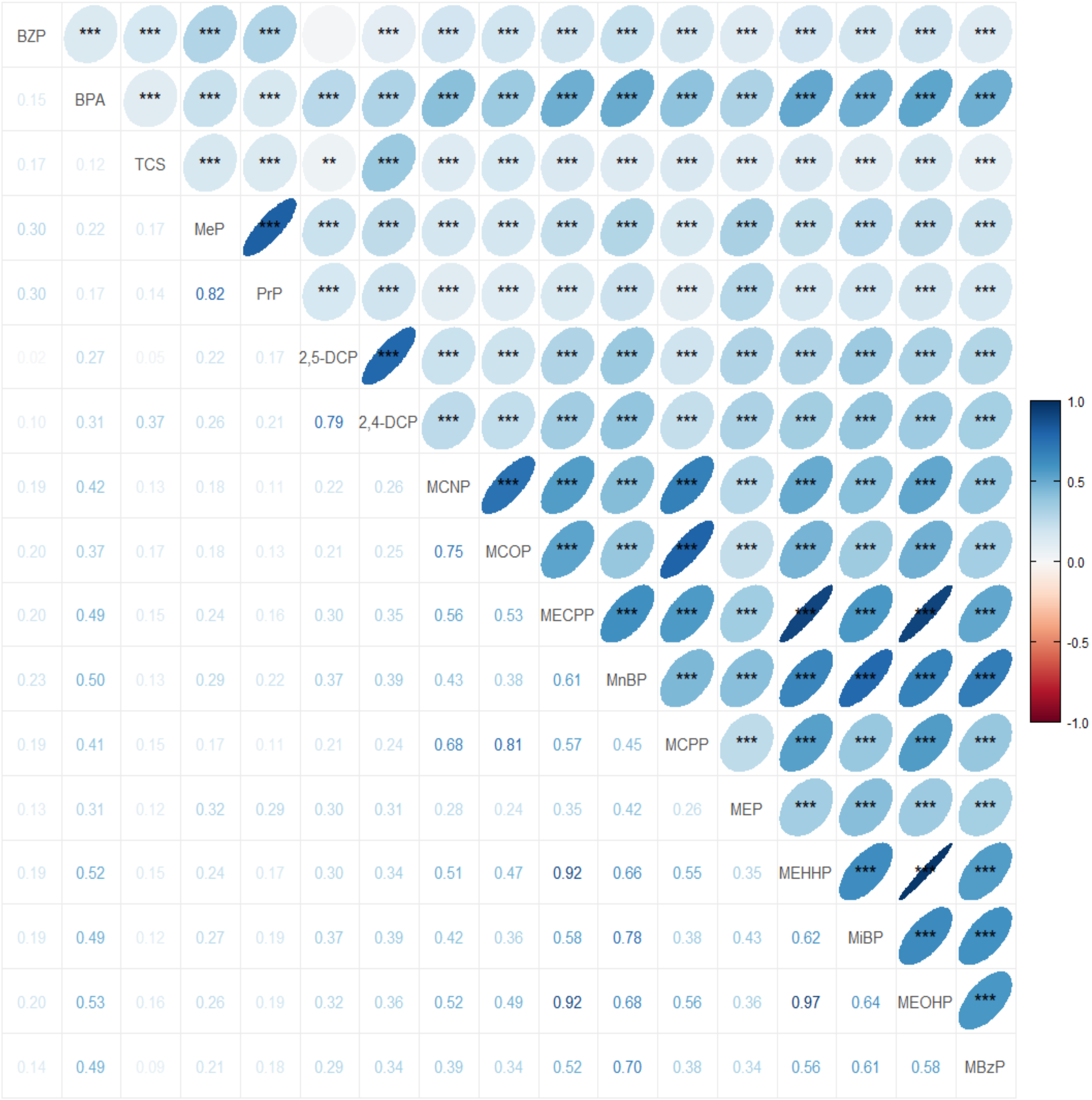

Figure S4. Bivariate exposure-outcome function between personal care product and plasticizing chemicals (PCPPCs) fixed on the top and short sleep duration among adults while fixing the PCPPCs on the right at 10th, 50th, and 90th percentiles in female.

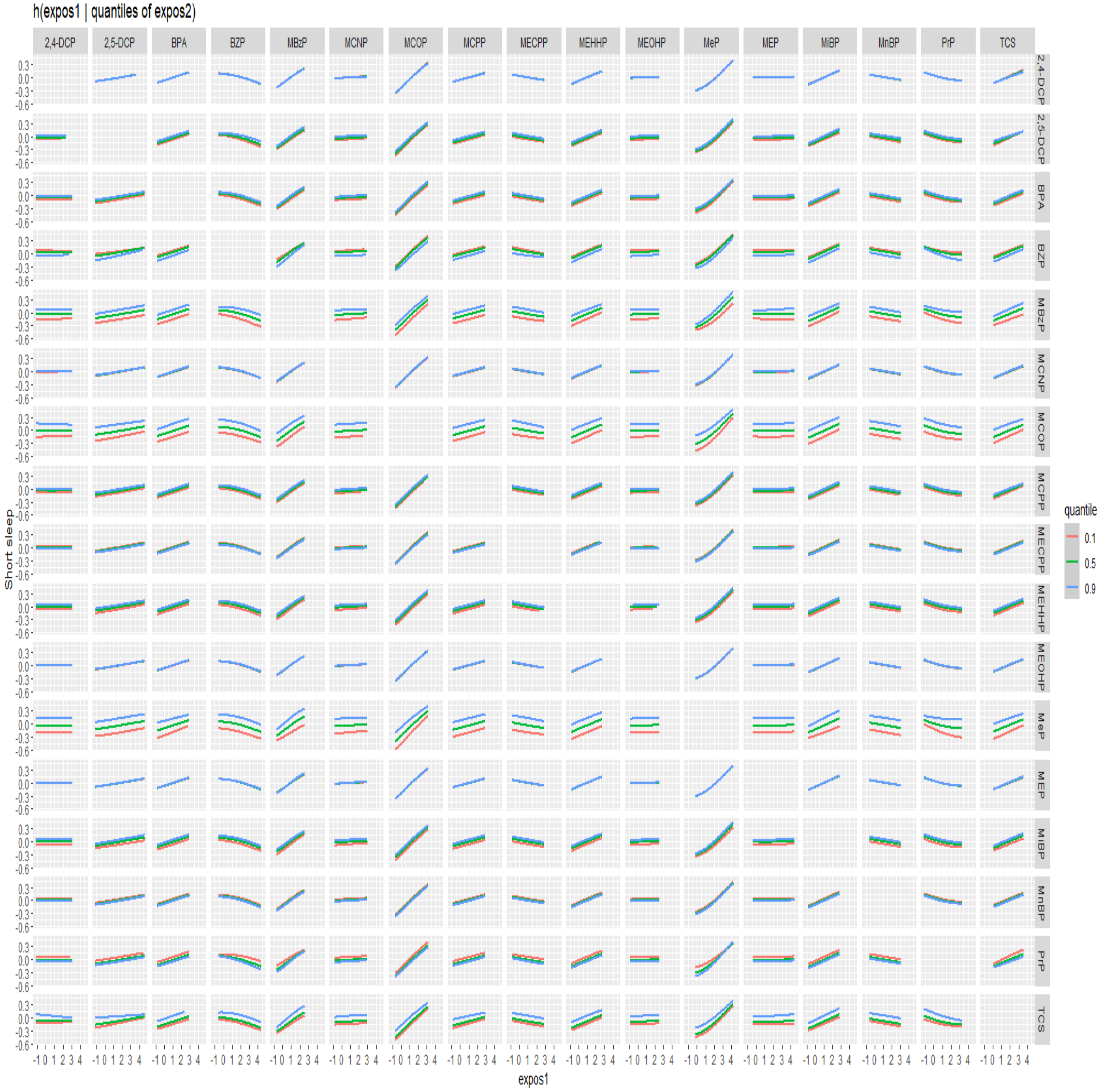

The Bayesian kernel machine regression model was adjusted for age, race, education, marital status, body mass index (BMI), waist circumference, family income to poverty ratio (PIR), food insecurity, physical activity, log cotinine, and log creatinine.

Figure S5. Bivariate exposure-outcome function between personal care product and plasticizing chemicals (PCPPCs) fixed on the top and short sleep duration among adults while fixing the PCPPCs on the right at 10th, 50th, and 90th percentiles in male.

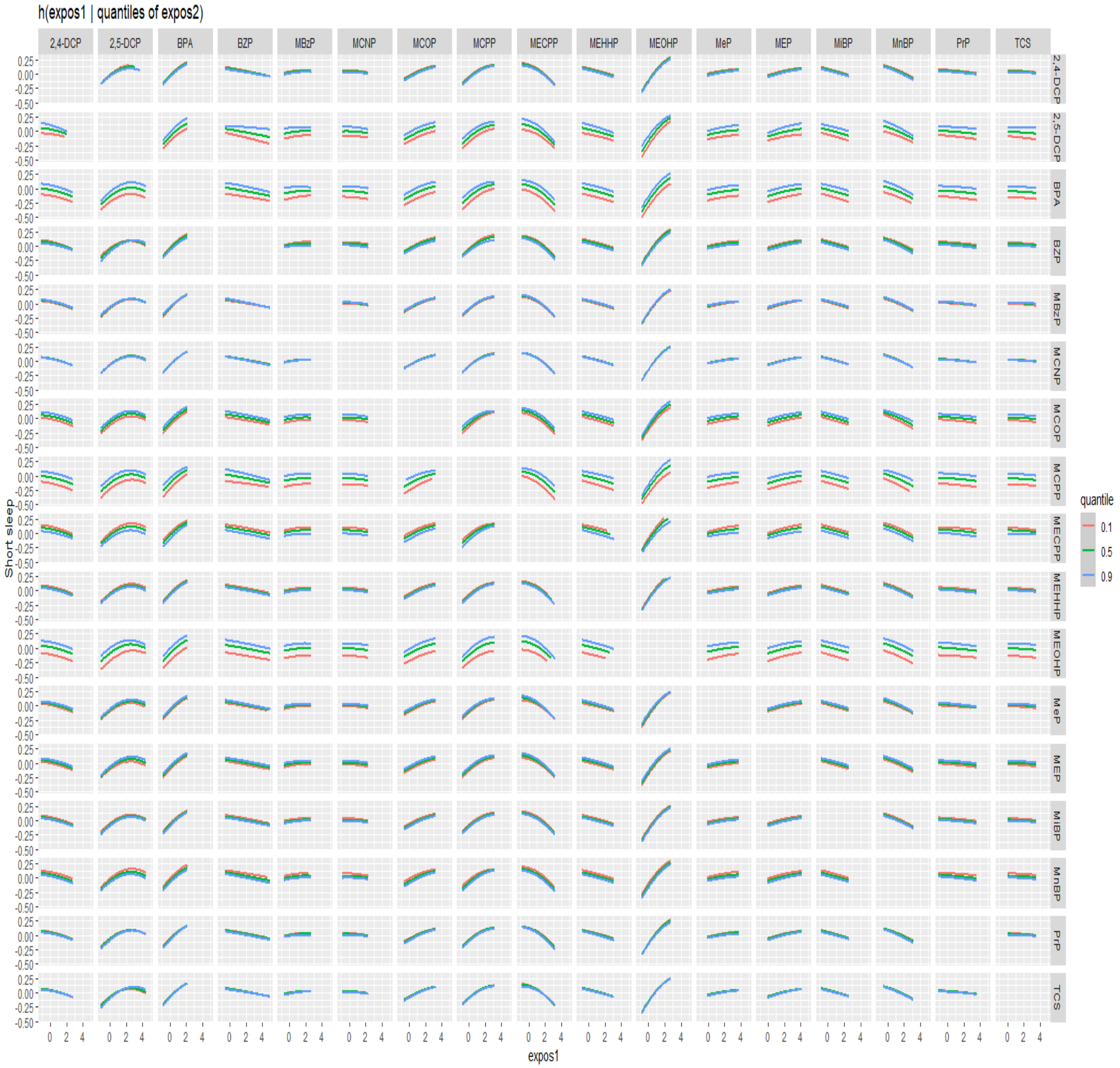

The Bayesian kernel machine regression model was adjusted for age, race, education, marital status, body mass index (BMI), waist circumference, family income to poverty ratio (PIR), food insecurity, physical activity, log cotinine, and log creatinine.

Figure S6. Bivariate exposure-outcome function between personal care product and plasticizing chemicals (PCPPCs) fixed on the top and short sleep duration among adults while fixing the PCPPCs on the right at 10th, 50th, and 90th percentiles in general obesity.

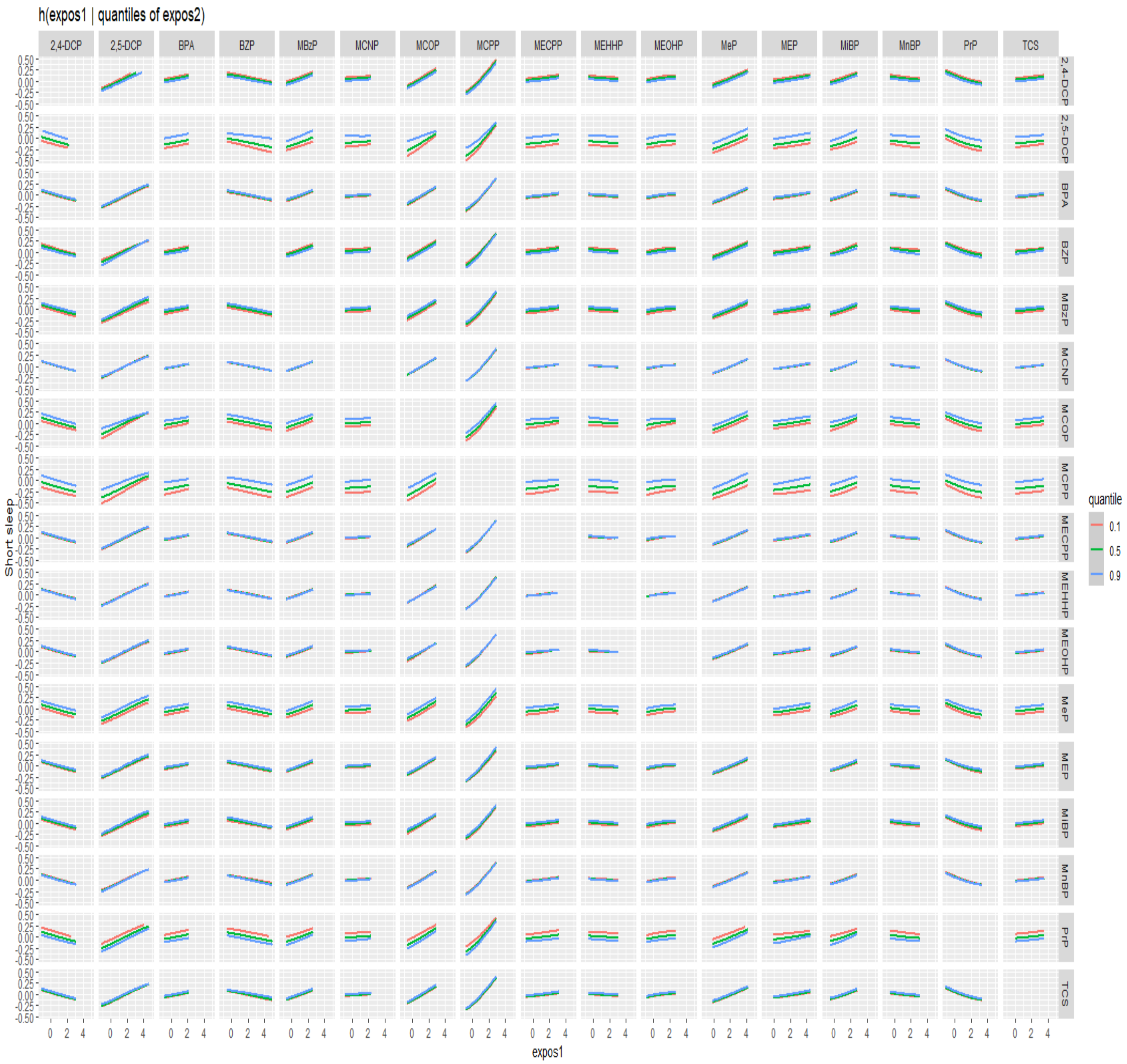

The Bayesian kernel machine regression model was adjusted for age, gender, race, education, marital status, waist circumference, family income to poverty ratio (PIR), food insecurity, physical activity, log_{10} cotinine, and log_{10} creatinine.

Figure S7. Bivariate exposure-outcome function between personal care product and plasticizing chemicals (PCPPCs) fixed on the top and short sleep duration among adults while fixing the PCPPCs on the right at 10th, 50th, and 90th percentiles in no general obesity.

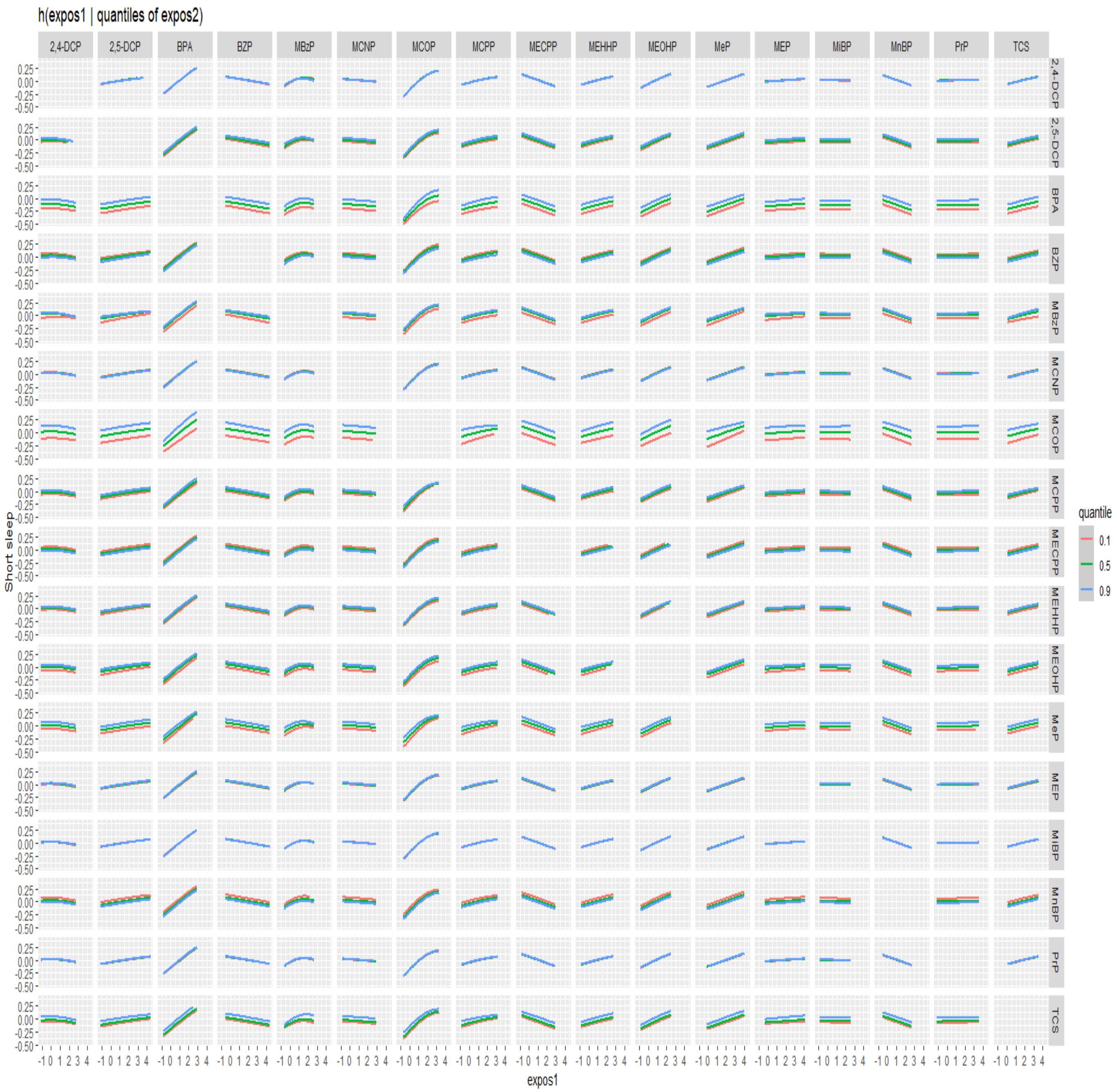

The Bayesian kernel machine regression model was adjusted for age, race, education, marital status, waist circumference, family income to poverty ratio (PIR), food insecurity, physical activity, $\log_{10}$ cotinine, and $\log_{10}$ creatinine.

Figure S8. Bivariate exposure-outcome function between personal care product and plasticizing chemicals (PCPPCs) fixed on the top and short sleep duration among adults while fixing the PCPPCs on the right at 10th, 50th, and 90th percentiles in abdominal obesity.

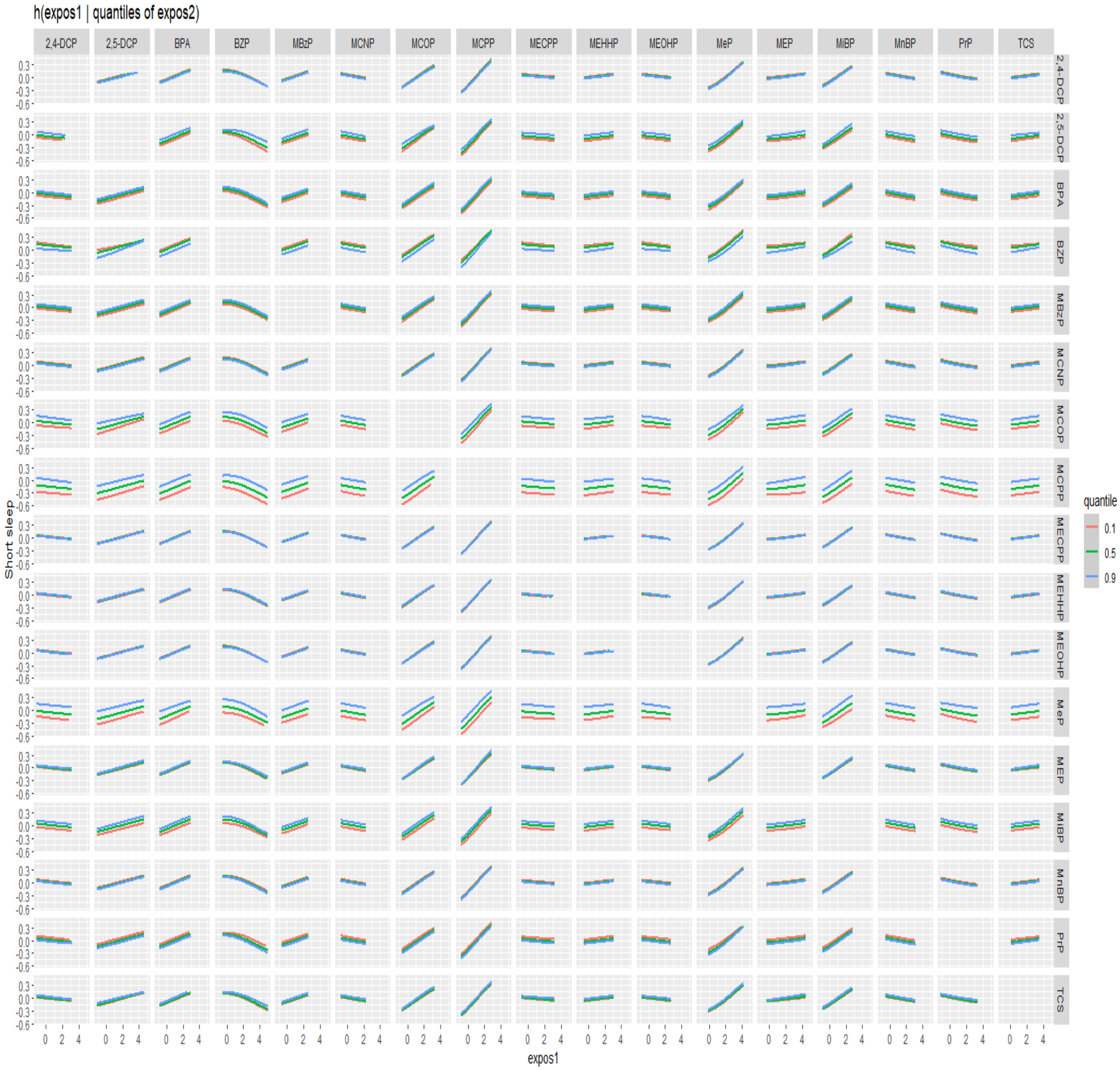

The Bayesian kernel machine regression model was adjusted for age, gender, race, education, marital status, body mass index (BMI), family income to poverty ratio (PIR), food insecurity, physical activity, log_{10} cotinine, and log_{10} creatinine.

Figure S9. Bivariate exposure-outcome function between personal care product and plasticizing chemicals (PCPPCs) fixed on the top and short sleep duration among adults while fixing the PCPPCs on the right at 10th, 50th, and 90th percentiles in no abdominal obesity.

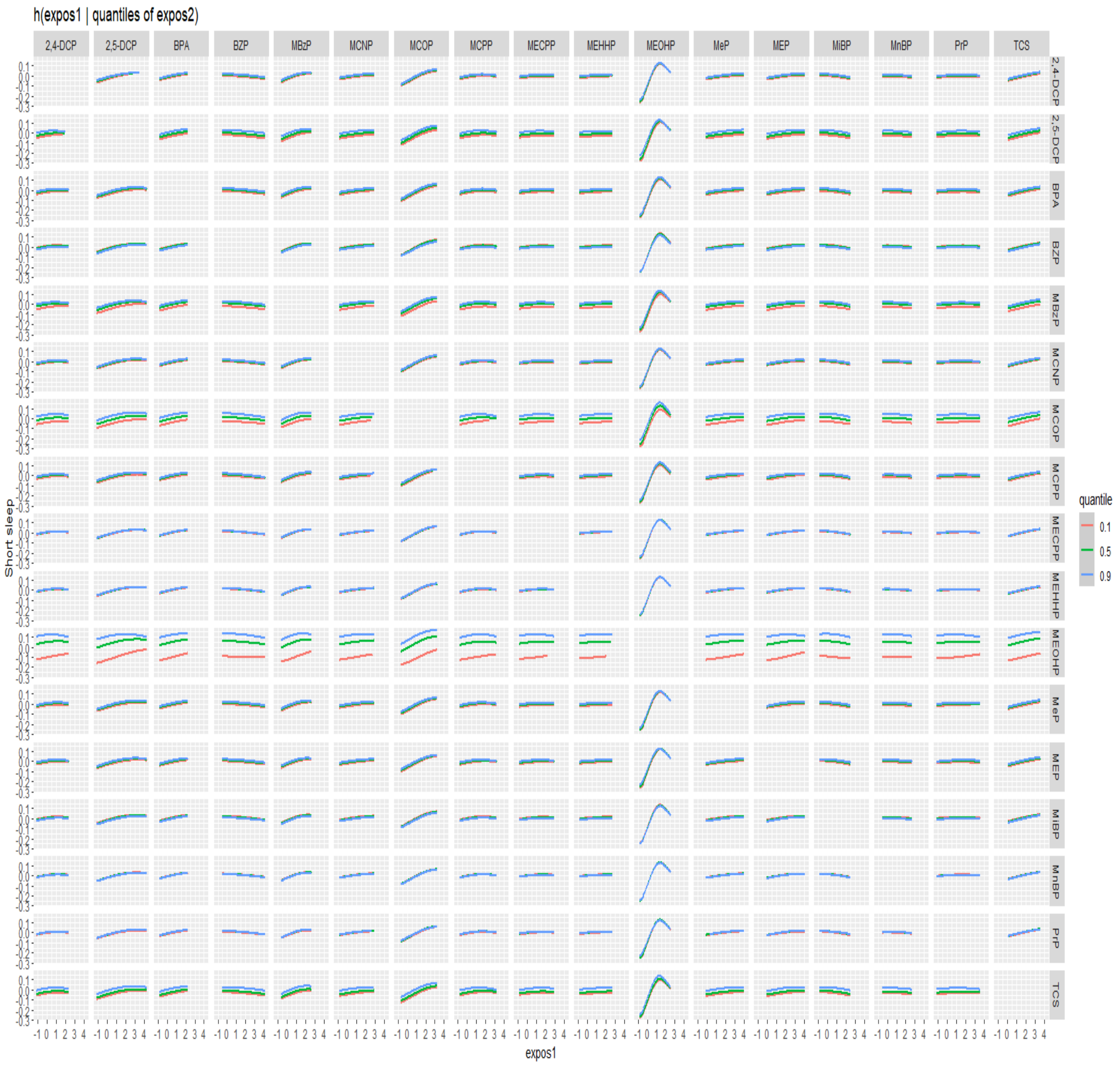

The Bayesian kernel machine regression model was adjusted for age, race, education, marital status, body mass index (BMI), family income to poverty ratio (PIR), food insecurity, physical activity, $\log_{10}$ cotinine, and $\log_{10}$ creatinine.

Figure S10. The mixture effect (95% confidence interval) of co-exposure to 17 personal care product and plasticizing chemicals (PCPPCs) on short sleep duration among adults by gender and obesity-specific after including 56 underweight participants in the primary analysis. A) Female. B) Male. C) General obesity. D) No general obesity. E) Abdominal obesity. F) No abdominal obesity.

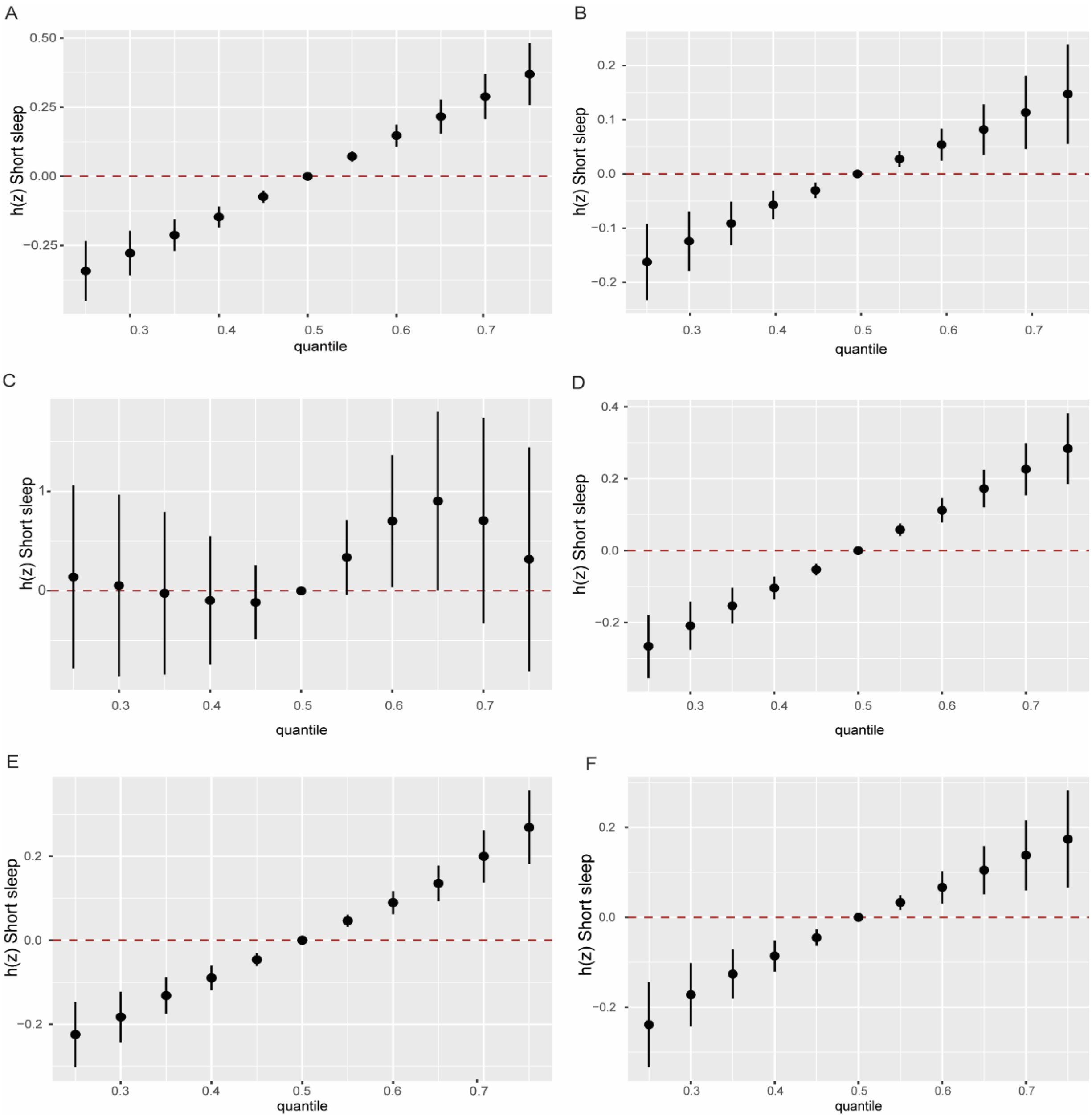

The Bayesian kernel machine regression model was adjusted for age, gender (not adjusted for female and male), race, education, marital status, body mass index (BMI) [Not adjusted for general obesity and no general obesity], waist circumference (Not adjusted for abdominal obesity and no abdominal obesity), family income to poverty ratio (PIR), food insecurity, physical activity, $\log_{10}$ cotinine and $\log_{10}$ creatinine.
